# Acupuncture for smoking cessation: an overview of systematic reviews

**DOI:** 10.3389/fpubh.2025.1677231

**Published:** 2026-01-15

**Authors:** Shun Fan, Jixuan Wang, Xiaoyu Wang, Haining Zhang, Huanan Li, Jianwu Wang, Wei Zhang, Jingui Wang

**Affiliations:** 1 First Teaching Hospital of Tianjin University of Traditional Chinese Medicine, Tianjin, China; 2 National Clinical Research Center for Chinese Medicine, Tianjin, China

**Keywords:** acupuncture, meta-analysis, overview, smoking cessation, systematic reviews

## Abstract

**Background:**

An increasing number of randomized controlled trials (RCTs) investigated various forms of acupuncture for smoking cessation; however, their findings remain inconsistent, and substantial controversy persists regarding both its efficacy and safety.

**Objectives:**

This overview aimed to synthesize the outcome evidence presented in existing systematic reviews (SRs) on acupuncture for smoking cessation, appraise the methodological quality of the included SRs, and re-evaluate the primary outcomes.

**Methods:**

A comprehensive literature search was conducted in eight databases from inception to December 31, 2024, with no language restrictions. Two independent reviewers screened the eligible studies. The methodological quality of the included SRs was assessed using A Measurement Tool to Assess Systematic Reviews 2 (AMSTAR 2). The Risk of Bias in Systematic Reviews (ROBIS) tool was used to evaluate potential bias. Reporting quality was assessed with the Preferred Reporting Items for Systematic Reviews and Meta-Analyses (PRISMA) 2020 checklist. The certainty of evidence from the SRs was rated using the Grading of Recommendations Assessment, Development, and Evaluation (GRADE) approach. After removing duplicate randomized controlled trials, we re-conducted meta-analysis for the primary outcomes using R software version 4.4.2 and TSA Viewer version 0.9.5.10, and performed trial sequential analysis (TSA) to examine the reliability and robustness of the findings.

**Results:**

This overview included 10 SRs, covering 74 RCTs. Using AMSTAR 2, ROBIS, and PRISMA 2020 for evaluation, we found that the methodological quality, risk of bias, and reporting quality of the 10 SRs were unsatisfactory. The quality of evidence evaluated by GRADE was mostly rated as low or critically low; however, acupuncture appeared to contribute to improved abstinence rates. In the updated meta-analysis, acupuncture showed superiority over sham acupuncture for short-term abstinence (RR = 1.37, 95% CI: 1.08, 1.73, *p* = 0.0092) and outperformed waiting list controls (*p* = 0.0204). However, no significant benefits were observed for long-term abstinence compared to sham, nor was acupuncture superior to nicotine replacement therapy or behavioral therapy. Furthermore, TSA indicated that the required information size was not reached, suggesting a risk of false-positive findings for the short-term benefit.

**Conclusion:**

Although the evidence for acupuncture in smoking cessation remains insufficient, acupuncture appears to improve short-term abstinence rates. However, substantial heterogeneity and methodological limitations were identified. TSA suggested a potential risk of false-positive findings, highlighting the need for further high-quality research.

**Systematic review registration:**

https://www.crd.york.ac.uk/PROSPERO/view/CRD42023473514, identifier CRD42023473514.

## 1 Introduction

The statement was made by the World Health Organization (WHO) that the population of smokers worldwide is approximately 1.245 billion by the year 2022 (1). Although global smoking prevalence dropped from 22.3% in 2007 to 16.4% in 2023, the absolute number of smokers remains high due to population growth (2). Tobacco use remains a significant global health challenge, causing over 7 million deaths annually, along with disabilities and long-term diseases related to tobacco (2). At present, pharmacotherapy is recommended by clinical guidelines as the most effective approach for smoking cessation (3, 4). However, the widespread application of pharmacological interventions—such as varenicline, bupropion, and nicotine replacement therapy (NRT)—remains limited due to concerns about adverse effects (e.g., nausea, insomnia, neuropsychiatric symptoms) (5), poor long-term adherence, and relatively low acceptability among smokers (6).

In light of these limitations, increasing attention was directed toward complementary and alternative therapies, particularly acupuncture. Acupuncture was employed as an auxiliary therapy for smoking cessation in multiple countries for over 40 years (7). Although guidelines suggest using acupuncture as an adjunctive or alternative therapy for smoking cessation (8), more high-quality research is necessary to substantiate its effectiveness. Nicotine dependence is primarily driven by the mesolimbic dopamine system (9). Experimental studies suggest that acupuncture modulates this pathway by stimulating the release of endogenous opioids (10, 11), which regulate dopamine release to alleviate withdrawal symptoms and reduce cravings (12–14).

Acupuncture has encompassed both traditional and non-traditional modalities. Traditional acupuncture employed filiform needles for stimulation, also known as manual acupuncture (MA). With the evolution of stimulation methods, electroacupuncture (EA), transcutaneous electrical acupoint stimulation (TEAS), and laser acupuncture (LA) were developed. However, individuals attempting smoking cessation may require more prolonged acupoint stimulation. As a result, certain studies have shifted focus to acupressure and intradermal needle retention. Continuous acupoint stimulation helped reduce the frequency of patient visits, thereby improving the convenience of the intervention and, to some extent, enhancing treatment adherence.

Currently, the number of clinical studies and systematic reviews (SRs) on acupuncture for smoking cessation has been steadily increasing. However, inconsistencies were observed among these SRs due to methodological flaws or insufficient reporting. It remained unclear whether the short-term cessation benefit of acupuncture was merely a placebo effect, and the certainty of the evidence was low. Therefore, this study comprehensively assessed the methodological and reporting quality of existing SRs on acupuncture for smoking cessation. It also aimed to offer recommendations for future systematic reviewers and clinical researchers.

## 2 Methods

### 2.1 Registration

The registration number CRD42023473514 was assigned to the protocol for this overview in PROSPERO. This overview adhered to the guidelines established by the Preferred Reporting Items for Overview of Systematic Reviews (15).

### 2.2 Search strategy

A comprehensive search was conducted by two independent researchers (HNZ and XYW) across eight databases: PubMed, EMBASE, Cochrane Library, Web of Science, Chinese National Knowledge Infrastructure, Chinese Biological Medicine, WanFang, and VIP database. The search covered the period from inception to December 31, 2024, without language restrictions. Manual searches of research registries and grey literature were also performed. Detailed search strategies for PubMed and EMBASE are provided in the Supplementary Table S1.

### 2.3 Inclusion and exclusion criteria

#### 2.3.1 Types of participants

Participants with severe illnesses or pregnancy were excluded. Studies focusing on acupuncture for alleviating withdrawal symptoms were excluded, as our focus was on the effect of acupuncture on the abstinence rate as a primary endpoint.

#### 2.3.2 Types of interventions

Acupuncture had to be the primary intervention. All types of acupuncture, including MA, EA, auricular acupressure (AACP), indwelling auricular needle (IAN), LA, and TAES, were included. Acupuncture could be used alone or in combination with different types of acupuncture, and pharmacotherapy or other therapies could be provided as background treatments.

#### 2.3.3 Types of comparators

Control group interventions included sham acupuncture (SA), waiting list (WL), no treatment (NT), or guideline-recommended treatments such as pharmacotherapy or behavioral therapy (BT). The control group was required to receive the same standard treatment as the intervention group. SRs comparing different types of acupuncture were excluded.

#### 2.3.4 Types of outcome measures

Only SRs that reported point abstinence rate as the primary outcome were eligible for inclusion, regardless of whether abstinence was biochemically verified. Secondary outcomes were extracted exclusively from these studies and included relief of short-term withdrawal symptoms, daily cigarette consumption, craving for cigarettes, exhaled carbon monoxide levels, blood cotinine levels, Fagerström Test for Nicotine Dependence (FTND), Minnesota Nicotine Withdrawal Scale (MNWS), Brief Questionnaire of Smoking Urges (QSU-Brief), Hamilton Depression Rating Scale (HAMD), Heaviness of Smoking Index (HSI), Beck Depression Inventory (BDI), and adverse event rates.

#### 2.3.5 Types of studies

SRs that included multiple randomized controlled trials (RCTs) were included. SRs based on non-RCTs, network meta-analyses, SRs without meta-analyses, umbrella reviews, overviews of SRs, editorials, review commentaries, and guidelines were excluded. When an author published multiple SRs, we retained all reviews that differed in the scope of interventions or included distinct sets of RCTs, and only excluded earlier versions when a later review was an updated version of the same review on the identical topic.

### 2.4 Study selection

The retrieved studies were imported into EndNote X20, and duplicates were subsequently identified and removed. Two reviewers (SF and XYW) independently screened the titles and abstracts of the studies and classified them as included, unclear, or excluded. Subsequently, studies classified as unclear or included underwent full-text review to determine their eligibility. Any discrepancies were resolved through discussion between the two reviewers or by consultation with a third reviewer (JGW) to reach a final decision.

### 2.5 Data collection

Based on data independently extracted from the SRs by two reviewers (JWW and JXW), followed by cross-verification, the following information was summarized: Initials of the first author, year of publication, number of RCTs, sample size, intervention details for both treatment and control groups, primary and secondary outcomes, methodological assessment tools, data analysis methods, and study conclusions.

In addition, we summarized data from all unique RCTs included in the SRs after removing duplicates. We extracted short-term and long-term point prevalence abstinence rates. Short-term was defined as the first follow-up visit after the end of treatment, occurring within 8 weeks from the initiation of smoking cessation. Long-term was defined as the final follow-up visit occurring between 6 and 12 months after the initiation of smoking cessation. These two timepoints were selected to evaluate the potential effects of acupuncture intervention from two distinct dimensions: (1) The short-term abstinence rate was used to assess whether acupuncture influenced the success rate during the acute withdrawal period, addressing the question: “Does acupuncture have an immediate harmful or beneficial effect?” (2) The long-term abstinence rate was used to assess whether acupuncture supports sustained smoking cessation, thereby evaluating its clinical value and long-term efficacy. We adhered to the guidance in Cochrane Handbook Chapter 23.3.4 regarding combining groups or splitting shared groups for independent comparisons (16).

### 2.6 Evaluation of review quality

The study primarily followed the Cochrane Handbook and associated methodologies for highly systematic evaluations and re-evaluations. The evaluation of quality comprised four dimensions: methodological quality, reporting quality, evidence quality, and risk of bias. Two investigators (SF and JXW) independently carried out the assessment. Disagreements were addressed through negotiation, and if that became necessary, handled by a third researcher (HNL).

#### 2.6.1 Methodological quality

A comprehensive assessment instrument for the evaluation of SRs of RCTs, an evaluation of the quality of the SRs that were included was carried out using the AMSTAR-2 tool (17). Seven of the 16 items assessed by this tool were considered crucial, including items 2, 4, 7, 9, 11, 13, and 15. If a review met all items or had just one non-critical item that was not met, it was considered to be of high quality. A moderate quality rating was given when more than one non-critical item was non-compliant. Low quality was indicated if there was non-compliance with one critical item, regardless of the non-critical items. Reviews were rated as critically low quality if more than one critical item was non-compliant, irrespective of the status of non-critical items.

#### 2.6.2 Risk of bias

The ROBIS tool operates through 3 distinct phases, facilitating the assessment of bias risk during the evaluation procedure, results, and conclusions (18). Responses to each question were categorized as “yes,” “probably yes,” “probably no,” “no,” or “no information.” The risk of bias in each domain was ultimately rated as “low risk,” “high risk,” or “uncertain.” If all key questions were answered with “yes” or “probably yes,” the bias risk in that domain was considered “low.” Conversely, if any key question was answered with “probably no” or “no,” there is a significant risk of bias. When the information was insufficient to pass judgment, the risk of bias was classified as “uncertain.”

#### 2.6.3 Report quality

The Preferred Reporting Items for Systematic Reviews and Meta-Analyses (PRISMA) 2020 statement is a widely used reporting guideline designed to assist in the identification, selection, evaluation, and synthesis of research (19). To evaluate the reporting quality of the included SRs, we applied the PRISMA 2020 checklist. Each item was evaluated with one of the three possible responses: “yes,” “partial yes,” or “no.” The completeness of each report was then calculated as the proportion of items answered with either “yes” or “partial yes” relative to the total number of applicable items. A report was considered sufficiently complete if more than 50% of the items received a “yes” or “partial yes” response, signifying an adequate level of reporting quality.

#### 2.6.4 Evidence quality

The GRADE (Grading of Recommendations, Assessment, Development, and Evaluation) instrument was used to evaluate the quality of evidence for each outcome measure in the SRs (20). This evaluation considered 5 factors that could potentially lead to a reduction in evidence quality: limitations, inconsistency, indirectness, imprecision, and publication bias. If no downgrade occurred, the evidence quality was classified as high. It was considered moderate if there was only 1 degradation, low if there were 2 downgrades, and extremely low if there were 3 or more downgrades. We not only rated the evidence synthesized in the systematic review but also rated the evidence obtained from the re-meta-analysis of the primary outcomes.

The following criteria were used to assess each downgrading factor:

(1) Limitations (Bias in Experimental Design): Downgraded when large biases, such as poor randomization, lack of blinding, or issues with distributive hiding, were present.
(2)Inconsistency (Heterogeneity): Downgraded when there was substantial variability between study results, indicated by a *p*-value smaller than 0.1 in the heterogeneity test or an *I*^2^ statistic greater than 50%.
(3)Imprecision (Wide Confidence Intervals or Small Sample Sizes): Downgrading was applied when the sample size was small (e.g., fewer than 500 participants) or when the confidence intervals were wide, specifically if they included the null line (*p* > 0.05), indicating substantial uncertainty in the effect estimate.
(4)Publication Bias: In line with a conservative approach to downgrading, publication bias was only considered when there were sufficient studies to assess its potential impact. Specifically, we did not apply downgrading for publication bias when fewer than 10 studies were included in the analysis, as the small number of studies may not provide reliable evidence of bias. When publication bias was assessed and identified through relevant tests (e.g., funnel plot asymmetry or Egger’s test), downgrading was applied accordingly.

### 2.7 Strategy for data synthesis

To evaluate the pooled effect size from the included SRs, we conducted a re-meta-analysis of the primary outcome using R software version 4.4.2. Two reviewers (WZ and SF) compiled data from all unique trials from the included SRs and performed data synthesis after removing duplicates. Risk ratios (RRs) with 95% confidence intervals (CIs) were used to express dichotomous outcomes. A fixed-effects model was applied when heterogeneity was not significant (*I*^2^ < 50%); otherwise, a random-effects model was used. When more than 4 studies were included, subgroup analyses were planned to explore potential sources of heterogeneity. Subgroup analyses were conducted based on acupuncture site, type of acupuncture, presence of stimulation in SA, combination with standard treatment, and biochemical verification of point prevalence abstinence. If subgroup analysis identified a potential source of heterogeneity, or if an insufficient number of studies precluded subgrouping, sensitivity analyses were performed using a leave-one-out approach to assess the robustness of results. To evaluate the robustness of acupuncture versus SA for short-term abstinence and to control for random errors due to limited sample size and repeated significance testing, trial sequential analysis (TSA) was performed using TSA Viewer version 0.9.5.10.

For each outcome, publication bias was assessed using Egger’s test and funnel plot visualization. When fewer than 10 studies were included, the power of Egger’s test to detect bias was limited, increasing the likelihood of false-negative results; thus, interpretations should be made with caution. When publication bias was detected, the trim-and-fill method was applied to adjust the pooled estimate and assess its impact. When zero-event data precluded the calculation of adjusted RR values using the trim-and-fill method, we relied on visual inspection of the funnel plots to assess asymmetry rather than reporting quantitatively adjusted estimates.

## 3 Results

### 3.1 Literature search and screening

**Figure 1:**
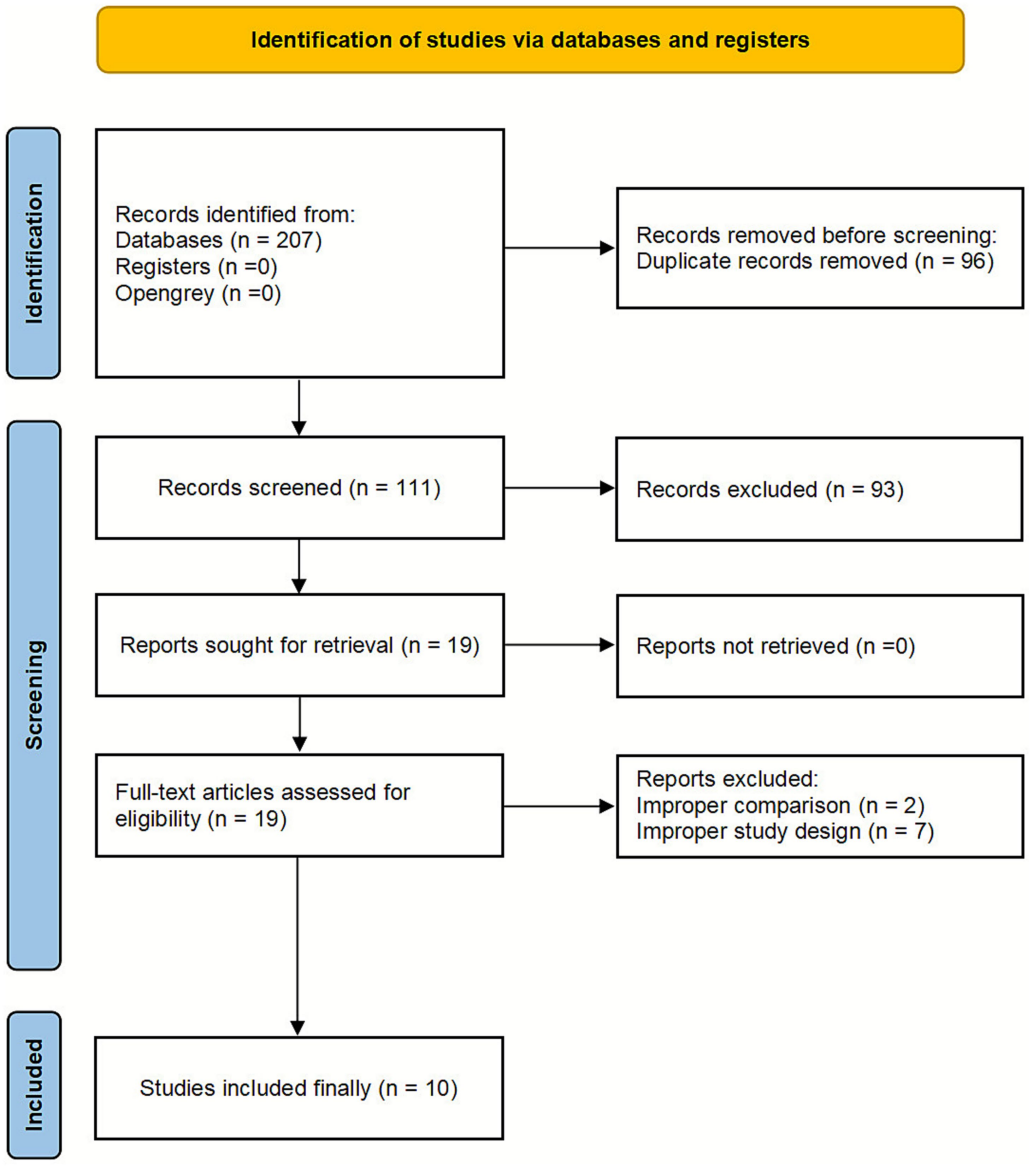
The process of literature selection.

Following the pre-formulated search strategy, an initial total of 207 original studies were retrieved. After eliminating duplicates with the software, 111 studies remained. A preliminary screening of titles and abstracts further reduced this number to 35 studies. Ultimately, after a thorough full-text review, 10 studies were included (21–30). The Supplementary Table S2 includes the studies excluded after full-text screening, along with detailed reasons for their exclusion. An illustration of the procedure for screening the literature was found in Figure 1.

### 3.2 Basic characteristics of the literature

This study included 10 SRs published between 1997 and 2024, with 2 published in 2012. Among the SRs, the number of RCTs was taken as an example. 7 were English-language studies (21, 24, 26–30) and 3 were Chinese-language studies (22, 23, 25). For the assessment of bias risk in RCTs, 1 paper utilized the Jadad score (28), 6 used the Cochrane Handbook recommended tool (21–24, 26, 27), and 2, due to their early publication date, employed other bias assessment methods (29, 30). Additionally, 1 paper used an evaluation manual based on CONSORT and STRICTA reporting guidelines (25). The intervention measures in the treatment groups primarily included MA, AACP, and IAN. The comparison groups were mainly given SA and BT. The fundamental features of the studies that were included were described in Table 1.

**Table 1:** Characteristics of the included SRs.

Study (author year)	Language	Number of studies (total sample)	Experimental group	Control interventions	Outcomes	Methodological evaluation tool	Main conclusion
Akram et al. (21)	English	4 (818)	LA	SA/BT	FTND/FR	Cochrane handbook	LA may be superior to BT in short-term abstinence rates.
Liu et al. (22)	Chinese	23 (2120)	MA/LA/ACE/FN	SA/BT/PT	AR/FTND/MNWS/QSU/HAMD/HSI	Cochrane handbook	Acupuncture shows no significant difference from PT in enhancing abstinence rates and may outperform BT.
Kuang et al. (23)	Chinese	16 (1976)	MA/LA/ACE/FN	SA/BT/PT	AR/FTND/MWNS	Cochrane handbook	AACP has a comparable abstinence rate to nicotine replacement therapy, and there is no statistical difference in terms of FTND and MNWS.
Zhang et al. (24)	English	14 (1435)	TEAS/LA/IAN/ACE	SA/BT/PT	AR/DCC/FTND/eCO	Cochrane handbook	AACP is more effective than SA or conventional therapy in the short term, and LA has a better long-term abstinence rate compared to SA.
Liu et al. (25)	Chinese	23 (1533)	MA/EA/AACP/TEAS/LA	SA/BT/PT	AR/DCC/CL/FTND/MNWS/BDI	Consort and Stricta	Compared with other therapies, acupuncture therapy can significantly improve the short-term abstinence rate, but it has no significant impact on the long-term abstinence rate.
White et al.(26)	English	30 (4581)	MA/LA/EA/AACP/TEAS/IAN	SA/PT/BT	AR	Cochrane handbook	AACP or LA offer no sustained benefits for smoking cessation lasting six months or more. EA is ineffective for smoking cessation.
Tahiri et al. (27)	English	6 (823)	MA/EA/LA	SA	AR	Cochrane handbook	Using EA or LA on the ear can effectively increase the smoking cessation rate compared to SA.
Cheng et al. (28)	English	20 (4155)	MA/EA/LA	SA/BT/PBO/WL	AR	Jaded	AACP is superior to SA in improving short-term abstinence rates, and acupuncture combined with BT is even more effective.
White (29)	English	12 (2013)	MA/EA	SA/PT/BT/PBO/WL	AR	Others	Acupuncture is more effective than WL treatment in improving short - term point - prevalence abstinence rates, but there is no significant difference compared with SA.
Ashenden et al. (30)	English	9 (4115)	MA/EA	SA/BT/PBO/WL	AR	Others	There is no significant difference between acupuncture and SA. Acupuncture does not show better efficacy than BT and nicotine replacement therapy.

MA, manual acupuncture; EA, electroacupuncture; AACP, auricular acupressure; IAN, indwelling auricular needle; TEAS, transcutaneous electrical acupoint stimulation; LA, laser acupuncture; ACE, acupoint catgut embedding; FN, fire needling; PT, pharmacotherapy (including nicotine replacement therapy, varenicline, and bupropion); BT, behavioral therapy; SA, sham acupuncture; WL, waiting list; PBO, placebo; AR, abstinence rate; DCC, Daily cigarette consumption; FTND, Fagerström Test for Nicotine Dependence; MNWS, Minnesota Nicotine Withdrawal Scale; QSU, Brief Questionnaire of Smoking Urges; HAMD, Hamilton Depression Rating Scale; HSI, Heaviness of Smoking Index; eCO, exhaled CO level; BDI, Beck Depression Inventory; FA, smoking cessation failure rate.

### 3.3 Results of quality assessment

#### 3.3.1 Methodological quality

The methodological quality of 8 SRs were evaluated as critically low quality, 1 SR as low, and 1 as moderate, which was evaluated employing the AMSTAR-2. The results were presented in Supplementary Table S3. Concerning critical items, 3 SRs (21, 24, 26) had protocols that were registered *a priori* (item 2). None of the SRs employed a comprehensive search strategy (item 4), and 2 SRs (24, 26) listed excluded studies with causes for their exclusion, as stated in item 7. Regarding evaluating the potential risk for bias, 6 SRs (21–24, 26, 27) considered the production of random sequences and the reporting of selective outcomes (item 9). For synthesizing study results, 7 SRs employed appropriate methods (item 11, except (27, 29, 30)), but only 2 SRs (21, 26) considered the risk of bias within each study during the discussion (item 13). As for publication bias (item 15), 3 SRs (21, 26, 28) provided thorough reports. For non-critical item evaluation, 2 SR (21, 24) listed funding sources for the included RCTs (item 10), and 1 SR (26) provided a detailed description of the studies that were included (item 8). 3 SRs (21, 24, 26) evaluated the potential influence that the risk of bias could have on the overall findings of each of the studies that were included (item 12). Although all SRs included RCTs, none explained their choice (item 3). 3 SRs (24, 26, 27) reported funding sources and disclosed potential conflicts of interest (item 16). 3 SRs (21, 26, 28) provided adequate explanations and justifications for any heterogeneity that was seen in the study findings (item 14).

### 3.4 Risk of bias

**Figure 2:**
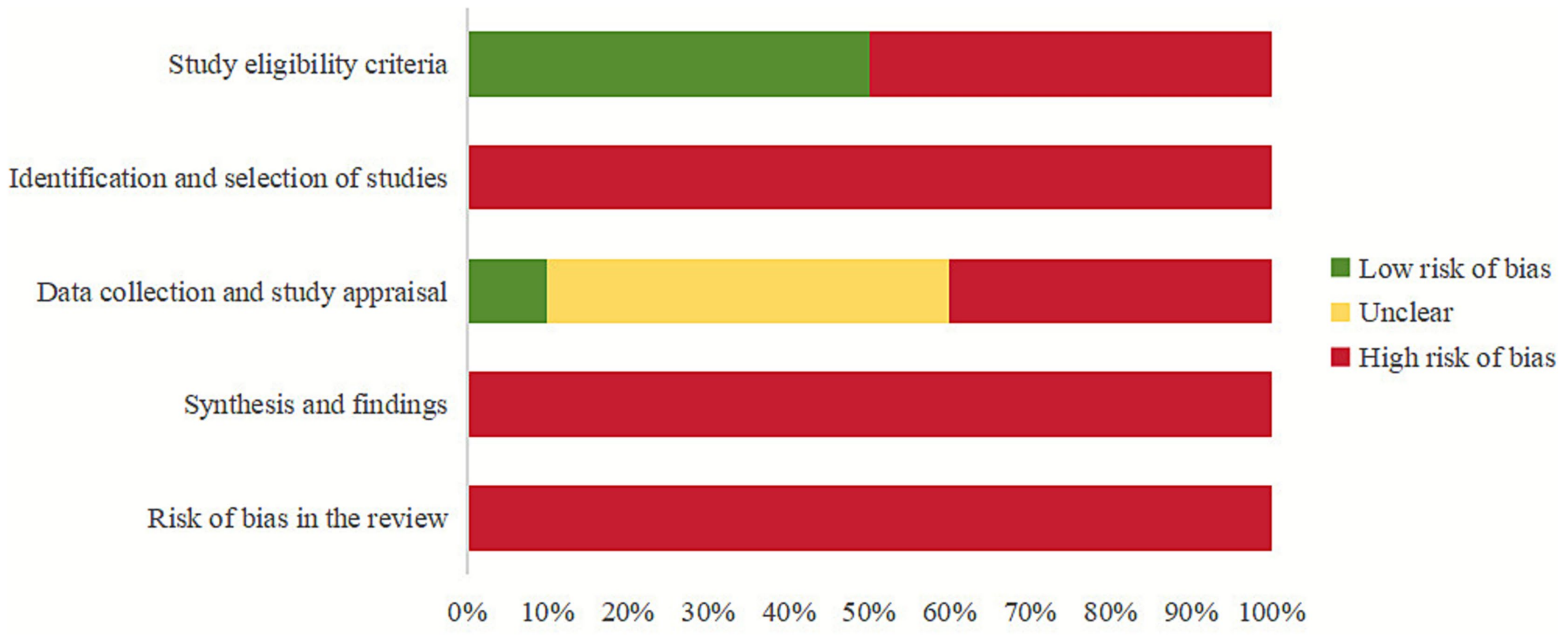
Risk of bias of the included SRs with the ROBIS tool.

According to the results of the risk of bias assessment for SRs, which was carried out with the use of the ROBIS tool, 5 SRs (21, 25, 27–29) were identified as being at high risk in Domain 1 of Phase 2, which is concerned with the eligibility for the study. In Domain 2, which evaluates study retrieval and screening, 100% of the 10 SRs were determined to be at a high risk. 1 SR (24) was deemed to have a low risk in Domain 3, which was concerned with the collection of data and the evaluation of the study. However, due to the risk of missing relevant literature during the retrieval process (as indicated in Domain 2), all SRs were rated as high risk in Domain 4, which covers synthesis and findings. Consequently, all SRs were ultimately rated as high risk in Phase 3, concerning the overall risk of bias in the review. The overall risk of bias judgments are summarized in Supplementary Table S4 and visualized in Figure 2, while the detailed assessment process and signaling question ratings are provided in Supplementary Table S5.

### 3.5 Reporting quality

The reporting quality, evaluated by the PRISMA 2020 guidelines, is summarized in Supplementary Table S6. The titles, introductions, and discussions of the included SRs were fully reported. However, several items were inadequately reported (<50%), including item 7, 22, 24, 26, and 27.

### 3.6 Evidence quality grading

The results of the evidence quality assessment, graded according to specific criteria, are detailed in Supplementary Table S7. All downgrading judgments were based on previously reported information. The SRs encompassed 64 outcome indicators. These indicators were evaluated based on 5 downgrading factors, namely, limitations (*n* = 64, 100%). inconsistency (*n* = 29, 45.3%), indirectness (*n* = 0, 0%), imprecision (*n* = 52, 81.3%) and publication bias (*n* = 3, 4.7%). In this study, none of the original RCTs were initially rated as high-quality evidence.

### 3.7 Effects of the intervention

#### 3.7.1 Comparison of acupuncture and sham acupuncture in short-term abstinence rates

**Figure 3:**
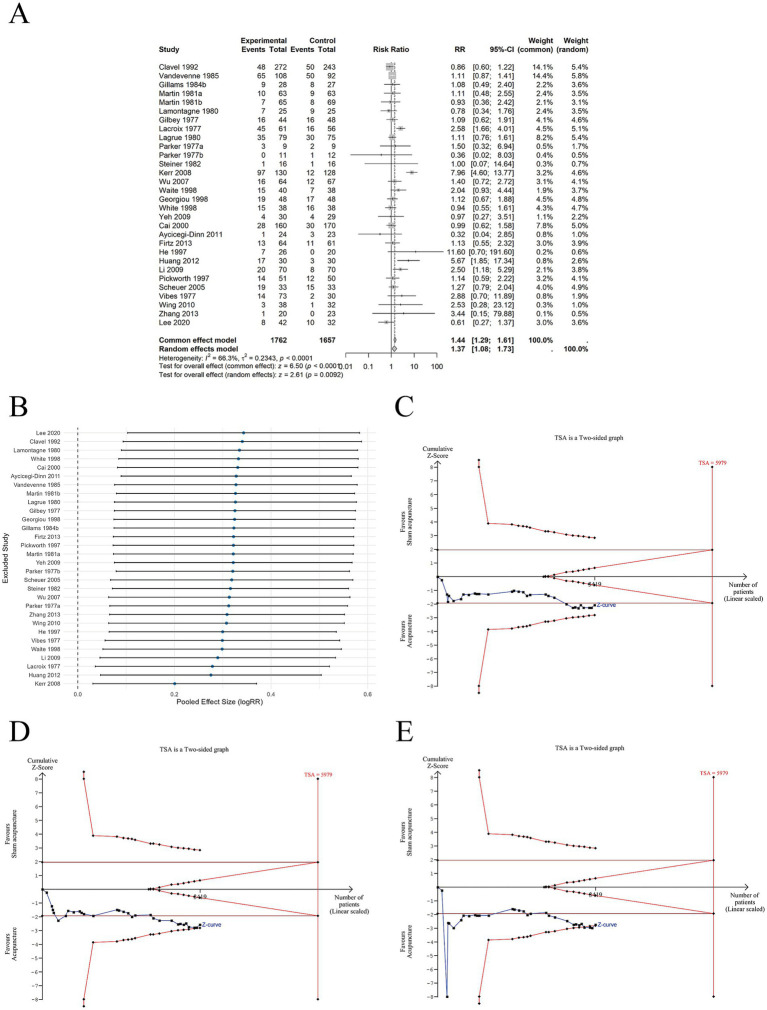
The comparison between acupuncture and SA for short-term abstinence. **(A)** Forest plot. **(B)** Sensitivity analysis. **(C)** Trial sequential analysis using the Sidik–Jonkman estimator. **(D)** Trial sequential analysis using the DerSimonian–Laird estimator. **(E)** Trial sequential analysis using the Biggerstaff–Tweedie estimator.

10 SRs (21–30) included 30 studies involving 3,419 participants, and the results showed that acupuncture was superior to SA in improving short-term abstinence rates (RR = 1.37, 95% CI: 1.08–1.73, *p* = 0.0092), with substantial heterogeneity observed among studies (*I*^2^ = 66.3%, *p* < 0.0001) (Figure 3A). We conducted subgroup analyses based on predefined variables, including acupuncture site, type of acupuncture, whether SA induced physiological stimulation, whether standard treatment was combined, and whether abstinence was biochemically verified; the results were presented in Table 2. The results revealed that heterogeneity decreased in some subgroups and increased in others; however, no statistically significant differences were observed between subgroups, suggesting that these variables did not account for the heterogeneity in the primary results. To further assess the robustness of the pooled results, we performed leave-one-out sensitivity analyses, which showed no substantial change in the overall effect estimate, indicating a high level of robustness (Figure 3B). In addition, we employed TSA to assess whether the current cumulative evidence has reached sufficient statistical power while controlling the risk of random errors. We set the type I error risk (*α*) at 0.05 and statistical power (1-*β*) at 0.80, using a two-sided test. Based on the findings of White et al. (26) in their Cochrane systematic review, we set the expected relative risk reduction at 22%. Using this value, we conducted TSA to estimate the effect in the intervention group. The abstinence rate in the intervention group was 30.5%, while in the control group, it was 25%. To ensure the robustness of the results, we employed three different methods to estimate between-study variance in the TSA: DerSimonian-Laird, Sidik-Jonkman, and Biggerstaff-Tweedie. The results showed that under all three methods, the cumulative Z-curves failed to cross the TSA-adjusted monitoring boundaries (Figures 3C–E), nor did they reach the required information size (RIS = 5,979), indicating a risk of false-positive findings and the need for further high-quality studies.

**Table 2:** Subgroup analysis on abstinence rate.

Characteristic	Subgroup analyses				Heterogeneity		Test for subgroup differences
	No. of studies	No. of subjects	RR (95% CI)	*p*-value	*I* ^2^	*p*	*p*-value
Short-term AR (Acupuncture vs. SA)							
Acupuncture site							0.0715
Body	5	953	1.28 (0.88, 1.88)	0.201	73.7	0.0043	
Auricular	19	1800	1.13 (0.94, 1.34)	0.192	0	0.6619	
Auricular + body	6	666	3.30 (1.32, 8.28)	0.0107	89.8	*p* < 0.0001	
Stimulation from sham acupuncture							0.7874
Yes	21	2,252	1.30 (1.06, 1.58)	0.0112	29.6	0.1004	
No	9	1,167	1.42 (0.77, 2.61)	0.265	85.7	*p* < 0.0001	
Validation of abstinence							0.1732
Yes	11	1,145	1.75 (1.06, 2.89)	0.0279	77.5	*p* < 0.0001	
No	19	2,274	1.20 (0.97, 1.49)	0.089	45.7	0.0161	
Standard treatment provided							0.1844
Yes	10	1,454	1.16 (0.87, 1.54)	0.321	59	0.009	
No	20	1965	1.57 (1.11, 2.22)	0.0111	69.3	*p* < 0.0001	
Type of acupuncture							0.1407
MA	6	1,068	1.18 (0.80, 1.74)	0.394	69.8	0.0054	
EA	2	99	0.91 (0.53, 1.55)	0.734	0	0.5536	
IAN	7	659	1.20 (0.88, 1.64)	0.243	0	0.8984	
AACP	4	327	1.50 (0.56, 4.01)	0.417	56.8	0.0735	
LA	2	588	2.80 (0.36, 21.52)	0.324	96.9	*p* < 0.0001	
MA + AACP	1	60	5.67 (1.85, 17.34)	0.00236	–	–	
EA + AACP	3	183	1.85 (0.97, 3.54)	0.0632	25.4	0.2619	
TEAS	5	435	1.15 (0.87, 1.53)	0.331	0	0.8286	
Long-term AR (Acupuncture vs. SA)							
Acupuncture site							0.0794
Body	1	515	0.61 (0.34, 1.10)	0.0986	–	–	
Auricular	10	1,418	0.89 (0.66, 1.19)	0.421	92.3	*p* < 0.0001	
Auricular + body	3	504	4.67 (0.83, 26.19)	0.08	0	0.5273	
Stimulation from sham acupuncture							0.6490
Yes	9	1,335	1.07 (0.71, 1.62)	0.738	36	0.1306	
No	5	1,102	1.44 (0.43, 4.86)	0.5522	89.1	*p* < 0.0001	
Validation of abstinence							0.5558
Yes	4	610	1.58 (0.73, 3.42)	0.25	30.6	0.2285	
No	10	1827	1.16 (0.58, 2.29)	0.677	79.9	*p* < 0.0001	
Standard treatment provided							0.2365
Yes	8	1,413	0.90 (0.61, 1.32)	0.594	13.4	0.3254	
No	6	1,024	1.79 (0.62, 5.19)	0.286	87.2	*p* < 0.0001	
Type of acupuncture							0.2217
AACP	1	74	0.76 (0.24, 2.41)	0.643	–	–	
EA	1	76	0.50 (0.05, 5.28)	0.565	–	–	
EA + AACP	2	124	9.42 (1.26, 70.66)	0.0291	0	0.9202	
IAN	4	446	1.20 (0.62, 2.32)	0.582	0	0.4294	
LA	2	613	3.76 (0.29, 48.90)	0.312	95.6	*p* < 0.0001	
MA	3	765	0.85 (0.46, 1.58)	0.608	59.1	0.0866	
TEAS	1	339	0.75 (0.49, 1,14)	0.181	–	–	

MA, manual acupuncture; EA, electroacupuncture; AACP, auricular acupressure; IAN, indwelling auricular needle; TEAS, transcutaneous electrical acupoint stimulation; LA, laser acupuncture.

#### 3.7.2 Comparison of acupuncture and sham acupuncture on long-term abstinence rates

**Figure 4:**
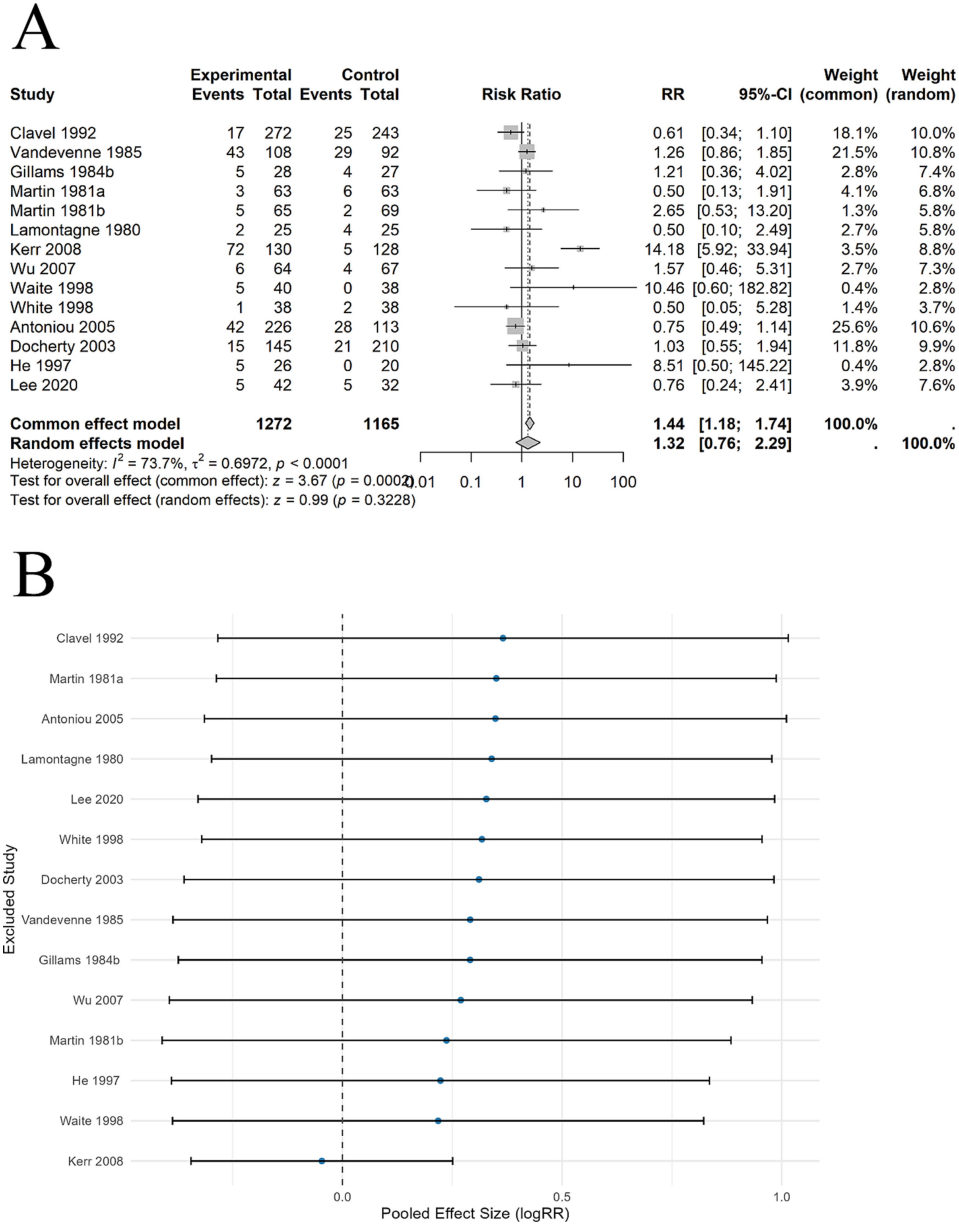
The comparison between acupuncture and SA for long-term abstinence. **(A)** Forest plot. **(B)** Sensitivity analysis.

9 SRs (21–24, 26–30) included 14 studies involving 2,437 participants. The pooled results showed no significant difference in long-term abstinence rates between acupuncture and SA (RR = 1.32, 95% CI: 0.76–2.29, *p* = 0.3228). Significant heterogeneity was observed (*I*^2^ = 73.7%, *p* < 0.0001) (Figure 4A). Predefined subgroup analyses were conducted, including acupuncture site, acupuncture type, whether SA induced stimulation, combination with standard therapy, and whether abstinence rates were biochemically verified. The results indicated that these subgroup factors were unlikely to be sources of heterogeneity. Table 2 presents the subgroup analysis results for long-term abstinence. To further assess the robustness of the pooled results, leave-one-out sensitivity analyses were conducted, which showed that the overall effect did not change significantly and consistently crossed the null line (logRR = 0), indicating high robustness of the results. However, no statistically significant effect was observed (Figure 4B).

#### 3.7.3 Comparison of acupuncture and nicotine replacement therapy on short-term abstinence rates

**Figure 5:**
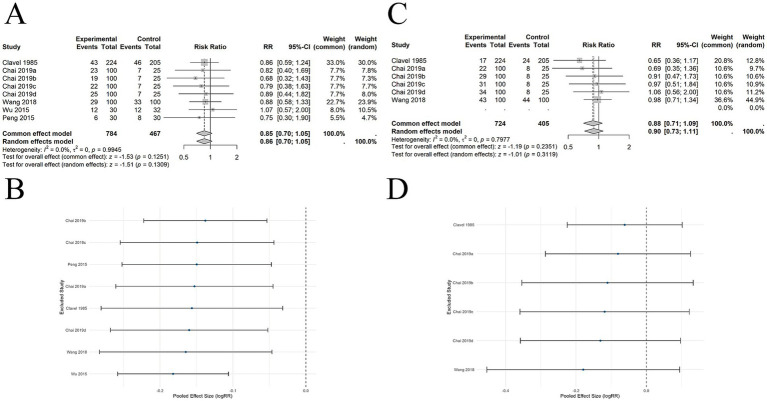
The comparison between acupuncture and NRT for abstinence rates: **(A)** Short-term abstinence: Forest plot; **(B)** Short-term abstinence: Sensitivity analysis; **(C)** Long-term abstinence: Forest plot; **(D)** Long-term abstinence: Sensitivity analysis.

8 SRs (22–26, 28–30) included 8 studies involving 1,251 participants. The pooled results demonstrated no significant difference in short-term abstinence rates between acupuncture and NRT (RR = 0.85, 95% CI: 0.70–1.05, *p* = 0.1251). No significant statistical heterogeneity was observed (*I*^2^ = 0%, *p* = 0.9945) (Figure 5A). Sensitivity analysis using a leave-one-out approach showed that the overall effect did not change significantly, consistently crossing the null line (logRR = 0), indicating high robustness of the results (Figure 5B).

#### 3.7.4 Comparison of acupuncture and nicotine replacement therapy on long-term abstinence rates

8 SRs (22–26, 28–30) included 6 studies involving 1,129 participants. The pooled results demonstrated no significant difference in long-term abstinence rates between acupuncture and NRT (RR = 0.88, 95% CI: 0.71–1.09, *p* = 0.2351) (Figure 5C). No significant statistical heterogeneity was observed (*I*^2^ = 0%, *p* = 0.7977). Sensitivity analysis using a leave-one-out approach showed that the overall effect did not change significantly, consistently crossing the null line (logRR = 0), indicating high robustness of the results (Figure 5D).

#### 3.7.5 Comparison of acupuncture and behavioral therapy on short-term abstinence rates

**Figure 6:**
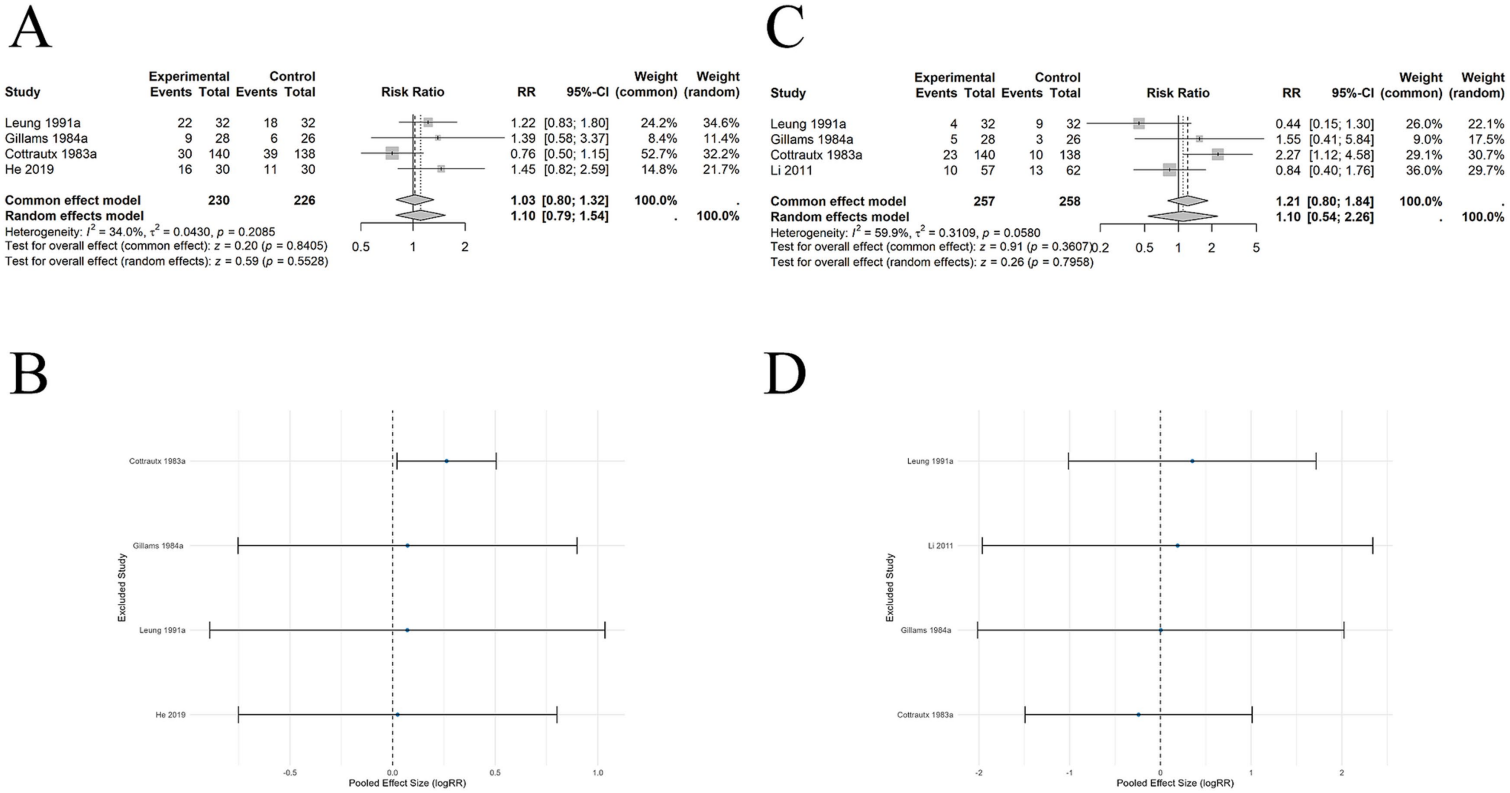
The comparison between acupuncture and BT for abstinence rates: **(A)** Short-term abstinence: Forest plot; **(B)** Short-term abstinence: Sensitivity analysis; **(C)** Long-term abstinence: Forest plot; **(D)** Long-term abstinence: Sensitivity analysis.

9 SRs (22–30) included 4 studies involving 456 participants. The pooled results demonstrated no significant difference in short-term abstinence rates between acupuncture and BT (RR = 1.03, 95% CI: 0.80–1.32, *p* = 0.8405). Low heterogeneity was observed among studies (*I*^2^ = 34.0%, *p* = 0.2085) (Figure 6A). Sensitivity analysis using a leave-one-out approach showed that after excluding some studies, the pooled effect did not cross the null line (logRR = 0), indicating slight instability of the results (Figure 6B).

#### 3.7.6 Comparison of acupuncture and behavioral therapy on long-term abstinence rates

7 SRs (24–30) included 4 studies involving 515 participants. The pooled results showed no significant difference in long-term abstinence rates between acupuncture and BT (RR = 1.10, 95% CI: 0.54–2.26, *p* = 0.7958). Moderate heterogeneity was observed among studies (*I*^2^ = 59.9%, *p* = 0.0580) (Figure 6C). Sensitivity analysis using a leave-one-out method demonstrated that the overall effect did not change significantly, consistently crossing the null line (logRR = 0), indicating robustness of the results (Figure 6D).

#### 3.7.7 Comparison of acupuncture and waiting list on short-term abstinence rates

**Figure 7:**
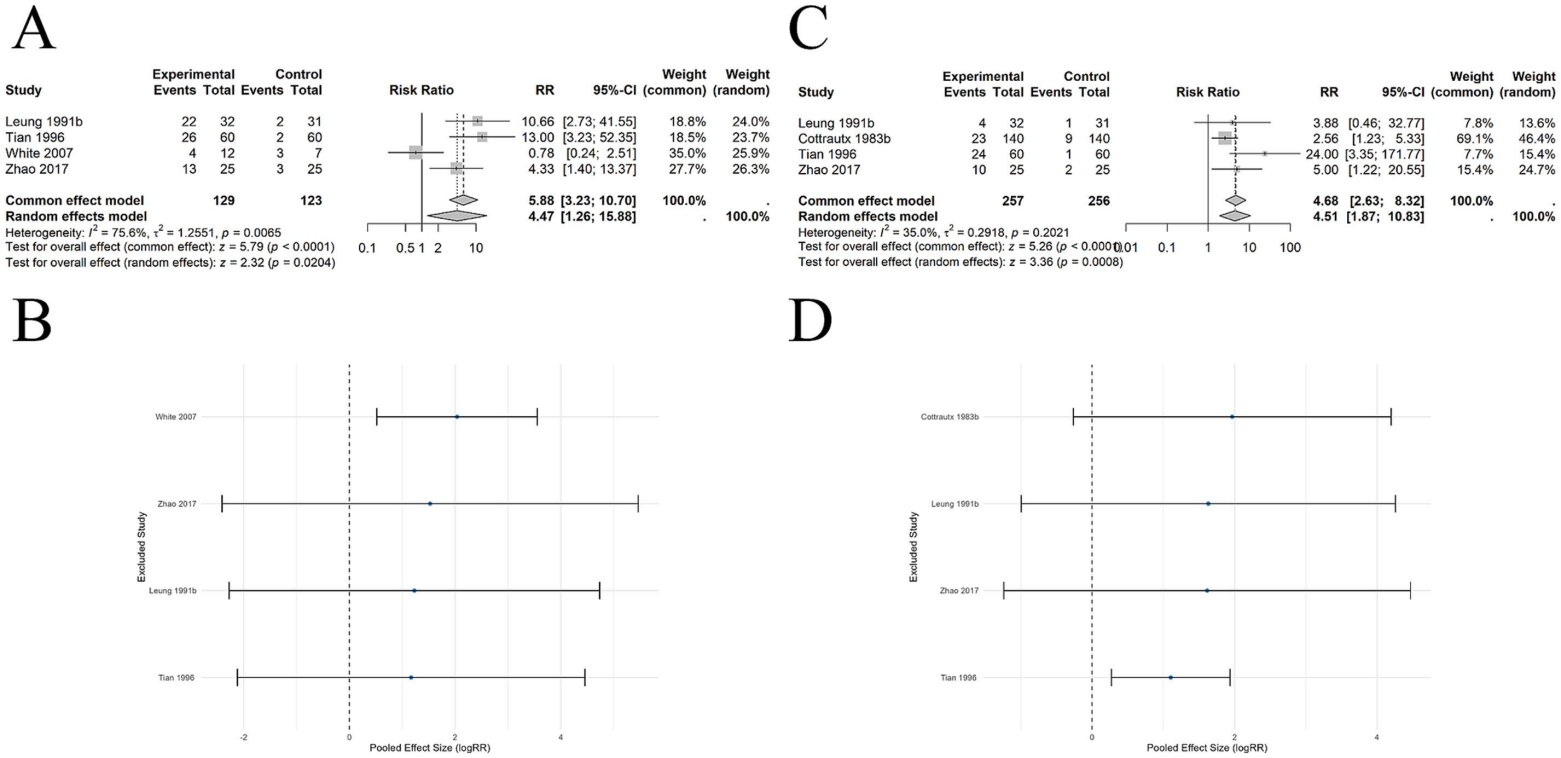
The comparison between acupuncture and WL for abstinence rates. **(A)** Short-term abstinence: Forest plot. **(B)** Short-term abstinence: Sensitivity analysis. **(C)** Long-term abstinence: Forest plot. **(D)** Long-term abstinence: Sensitivity analysis.

6 SRs (22, 24, 26, 28–30) included 4 studies involving 252 participants. The pooled results demonstrated a significant difference in short-term abstinence rates between acupuncture and WL (RR = 4.47, 95% CI: 1.26–15.88, *p* = 0.0204). Significant heterogeneity was observed among studies (*I*^2^ = 75.6%, *p* = 0.0065) (Figure 7A). Sensitivity analysis using a leave-one-out method showed that after excluding most studies, the pooled effect crossed the null line (logRR = 0), indicating less robust results (Figure 7B).

#### 3.7.8 Comparison of acupuncture and waiting list on long-term abstinence rates

7 SRs (22, 24–26, 28–30) included 4 studies encompassing 513 participants. The pooled results demonstrated a significant difference in long-term abstinence rates between acupuncture and WL (RR = 4.68, 95% CI: 2.63–8.32, *p* < 0.0001). Low heterogeneity was observed among studies (*I*^2^ = 35.0%, *p* = 0.2021) (Figure 7C). Sensitivity analysis using a leave-one-out approach showed that after excluding most studies, the pooled effect crossed the null line (logRR = 0), indicating less robust results (Figure 7D).

### 3.8 Adverse events

2 SRs (22, 24) reported adverse events and compared their incidence between groups. Adverse events in the AACP group included mild tenderness, dizziness, and itching. The primary adverse events in the TEAS group were pain. In the bupropion group, nausea and insomnia were reported. Other SRs did not report adverse events.

### 3.9 Publication bias

**Figure 8:**
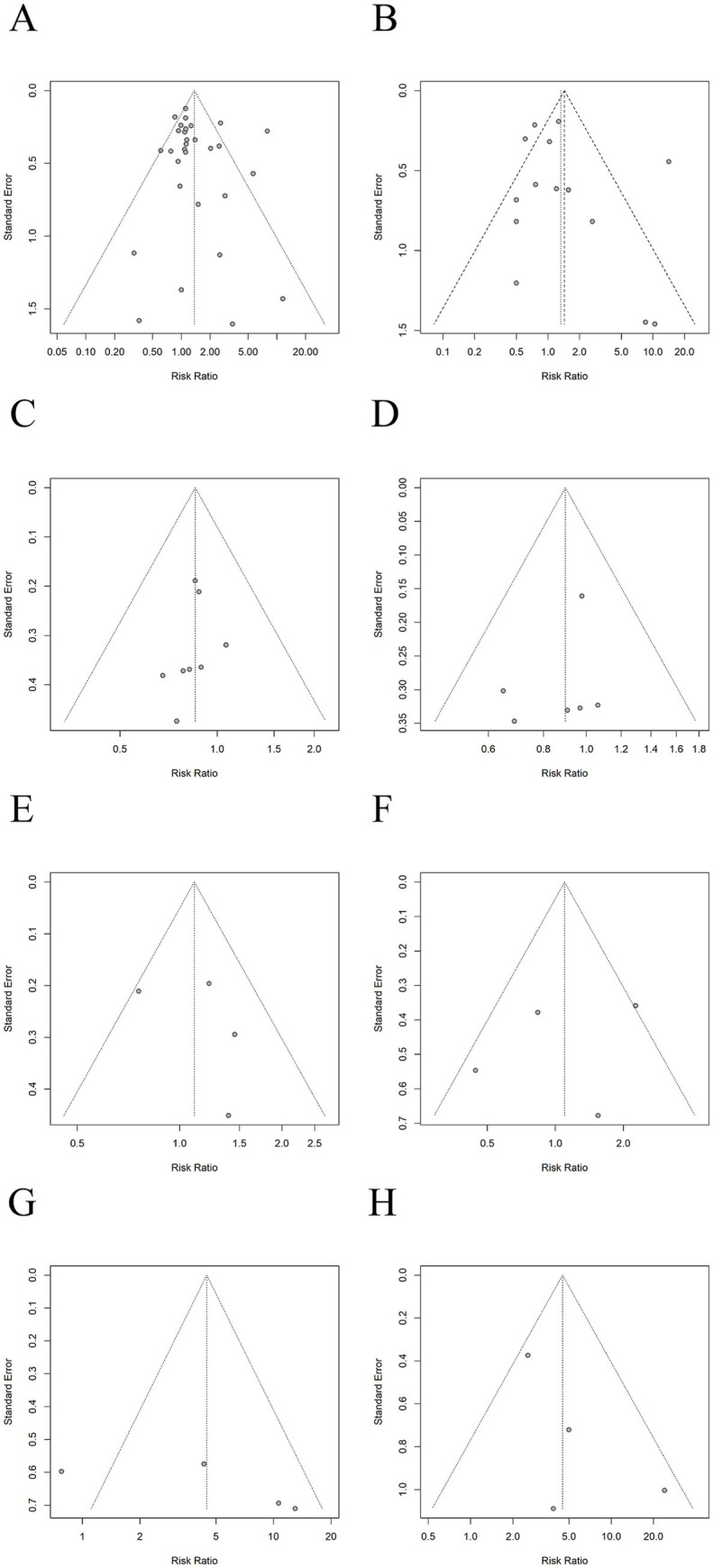
Egger’s test for assessment of potential publication bias (*p-*value). **(A)** Acupuncture vs. SA for short-term abstinence (*p* = 0.4158). **(B)** Acupuncture vs. SA for long-term abstinence (*p* = 0.4061). **(C)** Acupuncture vs. NRT for short-term abstinence (*p* = 0.4317). **(D)** Acupuncture vs. NRT for long-term abstinence (*p* = 0.3860). **(E)** Acupuncture vs. BT for short-term abstinence (*p* = 0.5539). **(F)** Acupuncture vs. BT for long-term abstinence (*p* = 0.6475). **(G)** Acupuncture vs. WL for short-term abstinence (*p* = 0.2924). **(H)** Acupuncture vs. WL for long-term abstinence (*p* = 0.2214).

We applied Egger’s test to evaluate the potential presence of publication bias in the aforementioned pooled results. No statistically significant publication bias was detected in these comparisons (all *p* > 0.05); detailed results are presented in the Supplementary Table S8. Visual inspection of the funnel plots (Figure 8) revealed no apparent asymmetry. However, for several outcomes, the number of studies was very limited, restricting test power. In the long-term abstinence comparison of acupuncture versus SA, and the short-term abstinence comparison of acupuncture versus waitlist control, a mild rightward asymmetry suggested possible publication bias favoring positive findings. Therefore, these results should be interpreted in conjunction with the funnel plots, and conclusions should be drawn cautiously.

### 3.10 GRADE assessment of updated meta-analysis

The GRADE evidence quality assessment for the outcomes indicates that most comparisons, including acupuncture versus SA, NRT, and BT, were downgraded to low or critically low quality due to serious limitations, inconsistency, and imprecision. The long-term acupuncture versus WL comparison was rated as moderate, as it showed less inconsistency and imprecision. These results are presented in Table 3. Overall, while acupuncture may show beneficial effects for AR, the evidence quality remains low, highlighting the need for further high-quality studies to strengthen the findings.

**Table 3:** Grade quality grading of updated meta-analysis.

Outcomes	Intervention vs. control	Number of studies (total sample)	Pooled effect size	*I*^2^ (%)	Limitations	Inconsistency	Indirectness	Imprecision	Publication bias	Quality of evidence
Short-Term AR	Acupuncture vs. SA	30 (3419)	RR = 1.37 (1.08, 1.73)	66	Serious	Serious	Not serious	Not serious	Not serious	L ⊕ ⊕ ⊖ ⊖^1,2^
Long-term AR	Acupuncture vs. SA	14 (2437)	RR = 1.32 (0.76, 2.29)	74	Serious	Serious	Not serious	Serious	Not serious	CL ⊕ ⊖ ⊖ ⊖^1,2,3^
Short-Term AR	Acupuncture vs. NRT	8 (1251)	RR = 0.85 (0.70, 1.05)	0	Serious	Not serious	Not serious	Serious	None	L ⊕ ⊕ ⊖ ⊖^1,3^
Long-term AR	Acupuncture vs. NRT	6 (1129)	RR = 0.88 (0.71, 1.09)	0	Serious	Not serious	Not serious	Serious	None	L ⊕ ⊕ ⊖ ⊖^1,2^
Short-Term AR	Acupuncture vs. BT	4 (456)	RR = 1.03 (0.80, 1.32)	34	Serious	Not serious	Not serious	Serious	None	L ⊕ ⊕ ⊖ ⊖^1,2^
Long-term AR	Acupuncture vs. BT	4 (515)	RR = 1.10 (0.54, 2.26)	60	Serious	Serious	Not serious	Serious	None	CL ⊕ ⊖ ⊖ ⊖^1,2,3^
Short-Term AR	Acupuncture vs. WL	4 (252)	RR = 4.47 (1.26, 15.88)	76	Serious	Serious	Not serious	Serious	None	CL ⊕ ⊖ ⊖ ⊖^1,2,3^
Long-term AR	Acupuncture vs. WL	4 (513)	RR = 4.68 (2.63, 8.32)	35	Serious	Not serious	Not serious	Not serious	None	M ⊕ ⊕ ⊕ ⊖^1^

^1^Downgraded if large biases such as poor randomization, lack of blinding, or issues with distributive hiding were present in the studies. ^2^Downgraded if there was significant variability between study results, indicated by a p-value smaller than 0.1 or an I^2^ statistic greater than 50%. ^3^Downgraded if the sample size was small, fewer than 500 participants, or if the confidence intervals were wide, particularly if they included the null line where p is greater than 0.05. ^4^Downgraded with caution. Publication bias was only considered when at least 10 studies were included and if relevant bias assessments, such as funnel plot asymmetry or Egger’s test, were conducted. No downgrading was applied when fewer than 10 studies were included or when publication bias testing was not performed. SA, sham acupuncture; WL, waiting list; BT, behavioral therapy; NRT, nicotine replacement therapy; AR, abstinence rate; CL, critically low; L, low; M, moderate.

## 4 Discussion

### 4.1 Summary of the main results

This study conducted a comprehensive reappraisal of existing SRs and meta-analyses on acupuncture for smoking cessation. The aim was to evaluate the evidence base regarding the safety and efficacy of acupuncture for smoking cessation. A total of 10 SRs were included, with more published in English than in Chinese. We employed four tools—AMSTAR-2, ROBIS, PRISMA, and GRADE—to comprehensively assess the methodological quality, risk of bias, reporting standards, and evidence certainty of the included reviews.

AMSTAR-2 assessments revealed that all included SRs had at least one critical methodological flaw. Only 1 SR (26) was rated as moderate quality, while all others were classified as “critically low” quality. The ROBIS assessment indicated that all SRs had a high risk of bias, suggesting that their conclusions may be systematically misleading. The PRISMA checklist showed that most studies had significant deficiencies in areas such as study selection flow, data extraction, and risk of bias description. Only two SRs (24, 26) had a completeness rate of over 70%. The GRADE assessment showed that most outcome indicators were rated as “low” or “critically low” in evidence certainty. The main reasons for downgrading were small sample sizes, unclear randomization processes, and inadequate implementation of blinding.

We conducted a new meta-analysis focusing on the primary outcomes—short-term and long-term point abstinence rates. The analysis indicated that acupuncture significantly improved short-term abstinence rates compared with SA. However, when compared with NRT or BT, the difference in short-term abstinence was not statistically significant. In terms of long-term abstinence, acupuncture showed superiority only compared to waitlist controls; no statistically significant differences were found when compared with other control groups.

To investigate the sources of potential heterogeneity, we conducted prespecified subgroup analyses, covering variables such as acupuncture modality, stimulation site, whether SA induced physiological stimulation, whether standard treatments were co-administered, and whether abstinence was biochemically validated. The results suggested that these factors were unlikely to be the main sources of heterogeneity. However, we acknowledge that clinical heterogeneity may still be a significant factor. Even after removing duplicate RCTs, differences in the definition of key concepts, such as “acupuncture,” “sham acupuncture,” and “successful smoking cessation,” across studies could contribute to the observed statistically high heterogeneity. This clinical variation is beyond our control and may be a fundamental driver of the heterogeneity observed in our meta-analysis. Although Georgiou et al. (31) found that FTND scores and baseline exhaled CO levels were associated with treatment efficacy, suggesting that smokers with lower nicotine dependence may benefit more from acupuncture, we were unable to conduct further subgroup analyses based on nicotine dependence levels due to limited data availability in the original studies.

Additionally, TSA was employed to assess the robustness of the results comparing acupuncture with SA in terms of short-term abstinence rates. Given the presence of significant heterogeneity, the use of the Biggerstaff-Tweedie method to estimate the RIS may be more appropriate. The results showed that although the cumulative Z-curve crossed the conventional significance boundary, it did not surpass the monitoring boundary set by TSA, indicating a risk of false-positive findings. Further analyses using the DerSimonian-Laird and Sidik-Jonkman methods yielded consistent conclusions, with no crossing of the TSA threshold, indicating that the current evidence remains insufficiently robust.

In summary, the generally low quality of existing SRs and primary studies, coupled with insufficient evidence strength, limits the clinical adoption of acupuncture as a primary smoking cessation therapy. Although some findings suggest acupuncture may have short-term efficacy in certain populations, there is currently no clear evidence to support its substitution for NRT or BT (3). Therefore, cautious application of acupuncture for smoking cessation is recommended in clinical practice. There is an urgent need for rigorously designed, high-quality, adequately powered multicenter RCTs in the future to further validate its clinical value and optimize intervention protocols.

### 4.2 Implications for further studies

This overview highlighted several issues that SR authors should take into consideration. The findings from the AMSTAR-2 tool indicated that the methodological quality of SRs needs improvement in the following areas. SRs should be prospectively registered in the Cochrane Library or PROSPERO and accompanied by a detailed study protocol to help reduce reporting bias. SRs should provide comprehensive search strategies, including searches of grey literature, conference abstracts, and clinical trial registries. A full search strategy should be provided for at least one major database, and any use of search filters should be reported to enhance transparency. A list of excluded studies, along with reasons for their exclusion, should be provided. SRs should fully describe the basic characteristics of the included studies, including study settings and follow-up durations. The Cochrane Risk of Bias tool (RoB 2.0) should be used to assess the risk of bias in the included studies. It is worth noting that the risk of bias should be assessed separately for each outcome. In addition, all sources of conflicts of interest should be disclosed.

The assessment using PRISMA 2020 revealed that most SRs demonstrated reporting deficiencies. It is recommended that structured abstracts be written and key elements such as sensitivity analyses, subgroup analyses, and GRADE summary tables be included. To align with internationally accepted reporting standards.

This study also assessed the risk of bias in the SRs using the ROBIS tool (32). We identified a relatively high risk of bias in domains 2 and 4 of phase 2. The issues in domain 2 were primarily related to inadequate search strategies, such as failure to include conference abstracts and registered studies. Domain 4 was characterized by improper methods of data synthesis, which may compromise the accuracy of effect estimates and suggest the possibility of publication bias. It is recommended to use funnel plots, sensitivity analyses, or statistical tests such as Begg’s and Egger’s tests when the number of studies is limited, to assist in evaluating result reliability.

According to the GRADE evidence profiles, most outcomes were rated as critically low quality, indicating inconsistency in results. The primary reasons for downgrading were limitations and imprecision, while inconsistency was a secondary factor. Based on the GRADE evidence, future RCTs should enhance the quality of study design, implementation, and reporting. The following considerations are proposed for future research:

(1) Future RCTs should employ central randomization systems or have independent statisticians generate random sequences with allocation concealed using the sequentially numbered, opaque, sealed envelopes (SNOSE). After implementing blinding in acupuncture trials, validated tools such as James or Bang’s blinding index should be used to assess the success of blinding, which is crucial for determining whether acupuncture’s effects are attributable to placebo. Some invasive SA methods may stimulate afferent nerve activity and induce physiological responses, making their effects resemble those of real acupuncture and reducing the inertness of the control group (33). Future studies should focus on designing low-impact SA controls while ensuring the feasibility and validity of blinding, as balancing these aspects remains a key challenge in acupuncture trial design.
(2) In explanatory RCTs, eligibility criteria should be defined based on nicotine dependence levels, as informed by prior research, to ensure homogeneity of the study population. Selecting participants using validated dependence assessment scales (e.g., the FTND Test) helps reduce individual variability in response to the intervention and thereby enhances the internal validity of the study. In contrast, for multicenter pragmatic RCTs that emphasize external validity, stratified randomization based on nicotine dependence level should be incorporated at the design stage. Alternatively, a prespecified plan for subgroup analysis should be included. This would help identify potential variations in the efficacy of acupuncture across different smoking populations. Thereby clarifies the target population and identifies subgroups most likely to benefit.

Notably, acupuncture for smoking cessation may hold a unique clinical value, particularly among individuals with co-occurring opioid dependence. Smoking prevalence in this population is significantly higher than in the general population; U.S. data indicate that their smoking rate is six times that of the general adult population, while their cessation success rate is considerably lower (34–38). A study by Lu et al. (39) found that acupuncture helped reduce methadone dosages during maintenance treatment, suggesting a potential regulatory effect on the neurobiological systems of individuals with opioid dependence. Therefore, acupuncture for smoking cessation warrants further investigation as an adjunctive intervention for this specific population. It may not only enhance smoking cessation outcomes but also potentially reduce opioid dosage requirements and issues related to tolerance, thereby contributing to broader health benefits.

(3) Regardless of the type of acupuncture used, reporting should adhere to the Consolidated Standards of Reporting Trials (CONSORT) statement and Standards for Reporting Interventions in Clinical Trials of Acupuncture (STRICTA) guidelines for transparent and standardized documentation. Common acupuncture modalities for smoking cessation include MA, AACP, IAN, and TEAS. For quantifiable acupuncture interventions, optimal parameters for TEAS or EA should be explored to assess the maximum therapeutic benefits for individuals attempting to quit smoking.
(4) In explanatory RCTs targeting smokers with mild nicotine dependence, superiority trials may use sham stimulation or waitlist controls. In trials involving smokers with opioid dependence, an add-on design may be adopted, in which acupuncture is added to standard treatment to explore the superiority of combination therapy.
(5) Regarding outcome selection, the abstinence rate as the primary endpoint should be prioritized. Continuous abstinence rate should be used to assess short-term treatment efficacy. Point prevalence abstinence may be used for long-term follow-up assessments. Biochemical validation should accompany abstinence assessment (40), using markers such as exhaled CO or serum cotinine. Thresholds for biochemical verification should be clearly defined. Smoking cessation is a progressive, multi-stage process toward “success.” Most smokers go through all or most of the cessation stages before achieving complete abstinence. Given the high relapse rate in tobacco dependence, long-term follow-up is essential in such clinical trials. With the emergence of the modern biopsychosocial model of medicine, patient-centered surrogate outcomes should be used, including measures of subjective experience, functional status, and quality of life, such as the SF-36. In long-term follow-up studies, hard endpoints related to disease risk among sustained quitters should be included. To comprehensively reflect the effectiveness of the intervention.

### 4.3 Strengths and limitations

First, this overview employed AMSTAR-2 and ROBIS to comprehensively assess the methodological quality and risk of bias in SRs on the effectiveness of acupuncture for smoking cessation. We determined the strength of the evidence using the GRADE approach. We excluded duplicate RCTs and conducted a new meta-analysis of the RCTs included in the SRs. Additionally, TSA was used to evaluate the short-term abstinence rate of acupuncture.

A limitation of this study was that the RCTs included in the meta-analysis were derived solely from the SRs included in the original studies, without conducting additional independent searches for newly published trials. As a result, recently published RCTs may not have been included, which could introduce publication bias and affect the robustness and generalizability of the findings. Although different reviewers independently evaluated and cross-checked the studies using AMSTAR-2 and ROBIS, discrepancies in the assessment may have arisen due to the subjective interpretation of individual scale items, which may have influenced the evaluation of study quality.

Additionally, the lack of data on nicotine dependence levels in the smoking cessation population limited our ability to conduct subgroup analyses on this variable, which could have provided further insights into the effects of treatment for different levels of nicotine dependence.

## 5 Conclusion

This overview synthesized findings from SRs on acupuncture interventions for smoking cessation, which included RCTs. Based on current evidence, acupuncture may be effective in improving short-term abstinence rates, particularly when compared to SA. Although prespecified subgroup analyses did not reveal significant sources of heterogeneity, we recognize that clinical heterogeneity may still play a role. Differences in the definitions of key concepts, such as intervention type, control measure, and outcome measures, could contribute to the observed heterogeneity, which is beyond our control. TSA indicated a potential risk of false-positive findings, likely due to the limited number of studies included in the analysis of acupuncture’s placebo effects for smoking cessation, which could result in an overestimation of the effect size and potentially undermine the reliability of the findings. To address these limitations, future high-quality studies should aim to standardize acupuncture protocols, increase sample size, and implement stricter blinding and randomization methods. Research should also ensure comprehensive reporting of outcome measures, explore various acupuncture approaches and control measures, and use larger datasets to surpass the monitoring boundaries of TSA-adjusted thresholds or RIS. These recommendations aim to enhance the reliability and generalizability of findings in future research.

## Data Availability

The original contributions presented in the study are included in the article/Supplementary material, further inquiries can be directed to the corresponding authors.

## References

[1] World Health Organization. WHO Global report on Trends in Prevalence of Tobacco Use 2000–2030. Geneva: World Health Organization (2024).

[2] World Health Organization. WHO Report on the Global Tobacco Epidemic, 2025: Warning about the Dangers of Tobacco. Geneva: World Health Organization (2025).

[3] Hays JT Ebbert JO Sood A. Treating tobacco dependence in light of the 2008 US Department of Health and Human Services clinical practice guideline. Mayo Clin Proc. (2009) 84:730–6. doi: 10.4065/84.8.730, 19648390 PMC2719526

[4] Shields PG Bierut L Arenberg D Balis D Cinciripini PM Davis J . Smoking cessation, version 3.2022, NCCN clinical practice guidelines in oncology. J Natl Compr Cancer Netw. (2023 Mar) 21:297–322. doi: 10.6004/jnccn.2023.0013, 36898367

[5] Rahimi F Massoudifar A Rahimi R. Smoking cessation pharmacotherapy; varenicline or bupropion? Daru. (2024) 32:901–6. doi: 10.1007/s40199-024-00539-6, 39264407 PMC11555175

[6] Aumann I Rozanski K Damm K Graf von der Schulenburg JM. Cost-effectiveness of pharmacological smoking cessation therapies - a systematic literature review. Gesundheitswesen. (2016) 78:660–71. doi: 10.1055/s-0035-1548852, 27784123

[7] Wang JH Wang M Liu SC Du XF Han M Liu JF . A bibliometric analysis of clinical study literature of traditional Chinese medicine therapies for smoking cessation. Tob Induc Dis. (2018) 16:15. doi: 10.18332/tid/86330, 31516415 PMC6659472

[8] Chen S Chang J Wang Y Liu Z Zhu H Yang J . Interpretation of WFAS clinical practice guideline on acupuncture and moxibustion: smoking cessation. Zhongguo Zhen Jiu. (2024) 44:1197–202. doi: 10.13703/j.0255-2930.20231226-k0005, 39401820

[9] Benowitz NL. Nicotine addiction. N Engl J Med. (2010) 362:2295–303. doi: 10.1056/NEJMra0809890, 20554984 PMC2928221

[10] Wang YY Yang JS Zhang O Li YC He LM Ma SQ . Progress on the research of acupuncture for smoking cessation in foreign and domestic. Zhongguo Zhen Jiu. (2013) 33:285–8. 23713328

[11] Qiao L Guo M Qian J Xu B Gu C Yang Y. Research advances on acupuncture analgesia. Am J Chin Med. (2020) 48:245–58. doi: 10.1142/s0192415x20500135, 32138535

[12] Wang YY Liu Z Chen F Sun L Wu Y Yang JS . Effects of acupuncture on craving after tobacco cessation: a resting-state fMRI study based on the fractional amplitude of low-frequency fluctuation. Quant Imaging Med Surg. (2019) 9:1118–25. doi: 10.21037/qims.2019.06.07, 31367566 PMC6629569

[13] Li QS Liu ZY Ma HJ Lü YY Fang YA Hou YZ . A preliminary study on the mechanism of ear-acupuncture for withdrawal of smoking. J Tradit Chin Med. (1987) 7:243–7. 3449704

[14] Cheng KJ. Neurobiological mechanisms of acupuncture for some common illnesses: a clinician's perspective. J Acupunct Meridian Stud. (2014) 7:105–14. doi: 10.1016/j.jams.2013.07.008, 24929454

[15] Bougioukas KI Liakos A Tsapas A Ntzani E Haidich AB. Preferred reporting items for overviews of systematic reviews including harms checklist: a pilot tool to be used for balanced reporting of benefits and harms. J Clin Epidemiol. (2018) 93:9–24. doi: 10.1016/j.jclinepi.2017.10.002, 29037888

[16] Higgins JPT Thomas J Chandler J Cumpston M Li T Page MJ . (editors). Cochrane Handbook for Systematic Reviews of Interventions version 6.5 (updated August 2024). London: Cochrane (2024). Available at: www.training.cochrane.org/handbook.

[17] Shea BJ Reeves BC Wells G Thuku M Hamel C Moran J . AMSTAR 2: a critical appraisal tool for systematic reviews that include randomised or non-randomised studies of healthcare interventions, or both. BMJ. (2017) 358:j4008. doi: 10.1136/bmj.j4008 28935701 PMC5833365

[18] Whiting P Savović J Higgins JP Caldwell DM Reeves BC Shea B . ROBIS: a new tool to assess risk of bias in systematic reviews was developed. J Clin Epidemiol. (2016) 69:225–34. doi: 10.1016/j.jclinepi.2015.06.005, 26092286 PMC4687950

[19] Page MJ McKenzie JE Bossuyt PM Boutron I Hoffmann TC Mulrow CD . The PRISMA 2020 statement: an updated guideline for reporting systematic reviews. BMJ. (2021) 372:n71. doi: 10.1136/bmj.n71 33782057 PMC8005924

[20] Guyatt GH Oxman AD Vist GE Kunz R Falck-Ytter Y Alonso-Coello P . GRADE: an emerging consensus on rating quality of evidence and strength of recommendations. BMJ. (2008) 336:924–6. doi: 10.1136/bmj.39489.470347.AD, 18436948 PMC2335261

[21] Akram Z Khairnar MR Naveen KPG Jadhav SK Kusumakar A Savitha PS. Effect of laser auricular acupuncture in tobacco smoking cessation: a systematic review and meta-analysis. Med Acupunct. (2024) 37:142. doi: 10.1089/acu.2023.0142 PMC12820658 41573020

[22] Liu ZY Chen SM Chang J Wang YY Yang JS. Acupuncture for treatment of tobacco withdrawal syndrome: systematic review and Meta-analysis. Chin Acupunct Moxibust. (2023) 43:575–83. doi: 10.13703/j.0255-2930.20220423-k0002, 37161812

[23] Kuang HJ Tang J Fu SS Cao Y Zhong F. Systematic evaluation of acupuncture and moxibustion for the treatment of tobacco dependence. Acta Chin Med. (2022) 37:661–72. doi: 10.16368/j.issn.1674-8999.2022.03.124

[24] Zhang YY Yu ZY Lan HD Liang SB Fang M Robinson N . Non-traditional acupuncture therapies for smoking cessation: a systematic review of randomized controlled trials. Eur J Integr Med. (2021) 47:101390. doi: 10.1016/j.eujim.2021.101390, 41468094

[25] Liu Z Wang Y Wu Y Yang J. Condition and effectiveness evaluation of acupuncture for smoking cessation. Chin Acupunct Moxibust. (2015) 35:851–7. doi: 10.13703/j.0255-2930.2015.08.029, 26571912

[26] White AR Rampes H Liu JP Stead LF Campbell J. Acupuncture and related interventions for smoking cessation. Cochrane Database Syst Rev. (2014) 2014:CD000009. doi: 10.1002/14651858.CD000009.pub4 24459016 PMC7263424

[27] Tahiri M Mottillo S Joseph L Pilote L Eisenberg MJ. Alternative smoking cessation aids: a meta-analysis of randomized controlled trials. Am J Med. (2012) 125:576–84. doi: 10.1016/j.amjmed.2011.09.028, 22502956

[28] Cheng HM Chung YC Chen HH Chang YH Yeh ML. Systematic review and meta-analysis of the effects of acupoint stimulation on smoking cessation. Am J Chin Med. (2012) 40:429–42. doi: 10.1142/S0192415X12500334, 22745061

[29] White AR Resch KL Ernst E. A meta-analysis of acupuncture techniques for smoking cessation. Tob Control. (1999) 8:393–7. doi: 10.1136/tc.8.4.393, 10629245 PMC1759757

[30] Ashenden R Silagy CA Lodge M Fowler G. A meta-analysis of the effectiveness of acupuncture in smoking cessation. Drug Alcohol Rev. (1997) 16:33–40. doi: 10.1080/09595239700186311, 16203409

[31] Georgiou AJ Spencer CP Davies GK Stamp J. Electrical stimulation therapy in the treatment of cigarette smoking. J Subst Abus. (1998) 10:265–74. doi: 10.1016/s0899-3289(99)00005-x, 10689659

[32] Perry R Whitmarsh A Leach V Davies P. A comparison of two assessment tools used in overviews of systematic reviews: ROBIS versus AMSTAR-2. Syst Rev. (2021) 10:273. doi: 10.1186/s13643-021-01819-x, 34696810 PMC8543959

[33] Lund I Näslund J Lundeberg T. Minimal acupuncture is not a valid placebo control in randomised controlled trials of acupuncture: a physiologist's perspective. Chin Med. (2009) 4:1. doi: 10.1186/1749-8546-4-1, 19183454 PMC2644695

[34] Jamal A King BA Neff LJ Whitmill J Babb SD Graffunder CM. Current cigarette smoking among adults - United States, 2005-2015. MMWR Morb Mortal Wkly Rep. (2016) 65:1205–11. doi: 10.15585/mmwr.mm6544a2, 27832052

[35] Guydish J Passalacqua E Tajima B Chan M Chun J Bostrom A. Smoking prevalence in addiction treatment: a review. Nicotine Tob Res. (2011) 13:401–11. doi: 10.1093/ntr/ntr048, 21464202 PMC3103720

[36] Nahvi S Richter K Li X Modali L Arnsten J. Cigarette smoking and interest in quitting in methadone maintenance patients. Addict Behav. (2006) 31:2127–34. doi: 10.1016/j.addbeh.2006.01.006, 16473476

[37] Richter KP Gibson CA Ahluwalia JS Schmelzle KH. Tobacco use and quit attempts among methadone maintenance clients. Am J Public Health. (2001) 91:296–9. doi: 10.2105/ajph.91.2.296, 11211643 PMC1446533

[38] Guydish J Passalacqua E Pagano A Martínez C Le T Chun J . An international systematic review of smoking prevalence in addiction treatment. Addiction. (2016) 111:220–30. doi: 10.1111/add.13099, 26392127 PMC4990064

[39] Lu L Chen C Chen Y Dong Y Chen R Wei X . Effect of acupuncture for methadone reduction: a randomized clinical trial. Ann Intern Med. (2024) 177:1039–47. doi: 10.7326/m23-2721, 38976882

[40] Velicer WF Prochaska JO Rossi JS Snow MG. Assessing outcome in smoking cessation studies. Psychol Bull. (1992) 111:23–41. doi: 10.1037/0033-2909.111.1.23, 1539088

