# Supplementary material for: Acupuncture for smoking cessation: an overview of systematic reviews
Source: Front Public Health. 2026 Jan 15;13:1677231. doi: 10.3389/fpubh.2025.1677231 (PMC12852475; doi:10.3389/fpubh.2025.1677231)
Supplement: Supplementary file 1 [file Supplementary_file_1.docx]

Supplementary Material

# Supplementary Table S1 Search strategy on two major databases

| **Database** | **Search strategy** |
| --- | --- |
| Pubmed | 1. "Smoking Cessation"[MeSH Terms]" OR "Tobacco use cessation"[MeSH Terms] 2. "Smoking Cessation"[Title/Abstract] OR "Smoking Cessations"[Title/Abstract] OR "stop$ smoking"[Title/Abstract] OR "giving up smoking"[Title/Abstract] OR "quit$ smoking"[Title/Abstract] OR "cease smoking"[Title/Abstract] OR "cigarette reduction"[Title/Abstract] OR "reduced cigarette"[Title/Abstract] OR "reduced tobacco consumption"[Title/Abstract] 3. #1 OR #2 4. 'Smoking Cessations'/exp 5. "acupuncture"[Title/Abstract] OR "Acupuncture Therapy"[Title/Abstract] OR "Auricular points"[Title/Abstract] OR "Ear Acupuncture"[Title/Abstract] OR "electroacupuncture"[Title/Abstract] OR "needle"[Title/Abstract] OR "needling"[Title/Abstract] OR "prick"[Title/Abstract] OR "pricking"[Title/Abstract] OR "quick puncture"[Title/Abstract] 6. #4 OR #5 7. "Meta-Analysis"[Publication Type] OR "Meta-Analysis as Topic"[MeSH Terms] 8. "Meta-Analysis"[Title/Abstract] OR "Clinical Trial Overviews"[Title/Abstract] OR "Overview, Clinical Trial"[Title/Abstract] OR "Systematic Reviews"[Title/Abstract] OR "Systematic Review"[Title/Abstract] OR "Cochrane review"[Title/Abstract] 9. #7 OR #8 10. #3 AND #6 AND #9 |
| Embase | 1. 'acupuncture'/exp 2. 'Acupuncture Therapy':ab,ti OR 'acupuncture, ear':ab,ti OR 'electroacupuncture':ab,ti OR 'acupuncture':ab,ti OR 'Acupuncture Therapy':ab,ti OR 'Auricular points':ab,ti OR 'Ear Acupuncture':ab,ti OR 'electroacupuncture':ab,ti OR 'needle':ab,ti OR 'needling':ab,ti OR 'prick':ab,ti 3. #1 OR #2 4. 'Smoking Cessations'/exp 5. 'smoking cessation':ab,ti OR 'tobacco use cessation':ab,ti OR 'Smoking Cessation':ab,ti OR 'stop$ smoking':ab,ti OR 'giving up smoking':ab,ti OR 'quit$ smoking':ab,ti OR 'cease smoking':ab,ti OR 'cigarette reduction':ab,ti OR 'reduced cigarette':ab,ti OR 'reduced tobacco consumption':ab,ti 6. 'meta analysis'/exp 7. 'Clinical Trial Overviews':ab,ti OR 'Overview, Clinical Trial':ab,ti OR 'Systematic Reviews':ab,ti OR 'Systematic Review':ab,ti OR 'Cochrane review':ab,ti 8. #7 OR #8 9. #3 AND #6 AND #9 |

# Supplementary Table S2 The Exclude study with reasons

| Title | Reasons |
| --- | --- |
| A systematic review of smoking cessation intervention studies in China | 1 |
| Acupuncture and smoking cessation, a review of the literature | 1 |
| Effect of acupuncture and auricular acupressure on smoking cessation Protocol of a systematic review and Bayesian network meta-analysis | 2 |
| Comparison between Acupuncture and Nicotine Replacement Therapies for Smoking Cessation Based on Randomized Controlled Trials: A Systematic Review and Bayesian Network Meta-Analysis | 2 |
| Acupuncture for smoking cessation: A protocol for a systematic review of randomized controlled trials | 3 |
| Effectiveness of stop smoking interventions among adults: Protocol for an overview of systematic reviews and an updated systematic review | 3 |
| A meta-analysis of ear-acupuncture, ear-acupressure and auriculotherapy for cigarette smoking cessation | 4 |
| Acupuncture for smoking cessation: A systematic review and meta-analysis of 24 randomized controlled trials | 4 |
| The effects of auricular acupuncture on smoking cessation may not depend on the point chosen--an exploratory meta-analysis | 5 |

The following are five reasons for literature exclusion:

1.Not systematic review or meta-analysis (n=2)

2.Systematic Review and Bayesian Network Meta-Analysis (n=2)

3.A protocol for systematic review and meta- analysis (n=2)

4.Acupuncture as an intervention in the control group (n=2)

5. Previous studies conducted by the same author (n=1)

# Supplementary Table S3 Results of the AMSTAR-2 assessments

| **Study** | **Q1** | **Q2** | **Q3** | **Q4** | **Q5** | **Q6** | **Q7** | **Q8** | **Q9** | **Q10** | **Q11** | **Q12** | **Q13** | **Q14** | **Q15** | **Q16** | **Overall quality** |
| --- | --- | --- | --- | --- | --- | --- | --- | --- | --- | --- | --- | --- | --- | --- | --- | --- | --- |
| Akram  (2024) (21) | Y | Y | N | PY | Y | Y | N | PY | Y | Y | Y | Y | Y | Y | Y | Y | L |
| Liu ZY  (2023) (22) | Y | N | N | PY | Y | Y | N | PY | Y | N | Y | N | N | N | N | N | CL |
| Kuang  (2022) (23) | Y | N | N | PY | Y | Y | N | PY | Y | N | Y | N | N | N | N | N | CL |
| Zhang  (2021) (24) | Y | Y | N | PY | Y | Y | Y | PY | Y | Y | Y | Y | N | N | N | Y | CL |
| Liu Z  (2015) (25) | Y | N | N | PY | Y | Y | N | PY | N | N | Y | N | N | N | N | N | CL |
| White  (2014) (26) | Y | Y | N | PY | Y | Y | Y | Y | Y | N | Y | Y | Y | Y | Y | Y | M |
| Tahiri  (2012) (27) | Y | N | N | PY | N | Y | N | PY | Y | N | N | N | N | N | N | Y | CL |
| Cheng  (2012) (28) | Y | N | N | PY | Y | Y | N | PY | N | N | Y | N | N | Y | Y | N | CL |
| White (1999) (29) | Y | N | N | PY | N | N | N | N | N | N | N | N | N | N | N | N | CL |
| Ashenden (1997) (30) | Y | N | N | PY | N | Y | N | PY | N | N | N | N | N | N | N | N | CL |
| Number of Y (%) | 10(100) | 3(30) | 0(0) | 0(0) | 7(70) | 9(90) | 2(20) | 1(10) | 6(60) | 2(20) | 7(70) | 3(30) | 2(20) | 3(30) | 3(30) | 4(40) |  |

**Abbreviations:** Y, yes; PY, partial Yes; N, no; M, moderate; L, low; CL, critically low.

# Supplementary Table S4 Summary of risk of bias judgments for included SRs using the ROBIS tool

| **Study** | **Study eligibility criteria** | **Identification and selection of studies** | **Data collection and study appraisal** | **Synthesis and findings** | **Risk of bias in the review** |
| --- | --- | --- | --- | --- | --- |
| Akram (2024)(21) | ☹ | ☹ | ？ | ☹ | ☹ |
| Liu ZY (2023)(22) | ☺ | ☹ | ？ | ☹ | ☹ |
| Kuang (2022)(23) | ☺ | ☹ | ？ | ☹ | ☹ |
| Zhang (2021)(24) | ☺ | ☹ | ☺ | ☹ | ☹ |
| Liu Z (2015)(25) | ☹ | ☹ | ☹ | ☹ | ☹ |
| White (2014)(26) | ☺ | ☹ | ？ | ☹ | ☹ |
| Tahiri (2012)(27) | ☹ | ☹ | ？ | ☹ | ☹ |
| Cheng (2012)(28) | ☹ | ☹ | ☹ | ☹ | ☹ |
| White (1999)(29) | ☹ | ☹ | ☹ | ☹ | ☹ |
| Ashenden (1997)(30) | ☺ | ☹ | ☹ | ☹ | ☹ |

**Notes:** ☺ =low risk; ☹ =high risk; ？=unclear risk.

# Supplementary Table S5 Detailed domain-level ratings and assessment process using the ROBIS tool

| **Section/topic** | **Akram (2024) (21)** | **Liu ZY (2023) (22)** | **Kuang (2022) (23)** | **Zhang (2021) (24)** | **Liu Z (2015) (25)** | **White (2014) (26)** | **Tahiri (2012) (27)** | **Cheng (2012) (28)** | **White (1999) (29)** | **Ashenden (1997) (30)** |
| --- | --- | --- | --- | --- | --- | --- | --- | --- | --- | --- |
| **Phase 1 Assessing relevance** | L | L | L | L | L | L | L | L | L | L |
| **population(s)** | Y | Y | Y | Y | Y | Y | Y | Y | Y | Y |
| **Intervention(s)** | Y | Y | Y | Y | Y | Y | Y | Y | Y | Y |
| **Comparator(s)** | Y | Y | Y | Y | Y | Y | Y | Y | Y | Y |
| **Outcome(s)** | Y | Y | Y | Y | Y | Y | Y | Y | Y | Y |
| **Phase 2-domain 1: study eligibility criteria** | H | L | L | L | H | L | H | H | H | L |
| **2.1.1** | Y | PY | PY | Y | PY | Y | PY | PY | PY | PY |
| **2.1.2** | Y | Y | Y | Y | Y | Y | Y | Y | Y | Y |
| **2.1.3** | Y | Y | Y | Y | N | Y | N | N | N | Y |
| **2.1.4** | Y | Y | Y | Y | PN | Y | PN | Y | Y | Y |
| **2.1.5** | N | Y | Y | Y | Y | Y | Y | N | PN | Y |
| **Phase 2-domain 2: identification and selection of studies** | H | H | H | H | H | H | H | H | H | H |
| **2.2.1** | N | N | N | N | N | Y | N | N | N | PY |
| **2.2.2** | N | N | N | Y | N | PN | N | N | Y | Y |
| **2.2.3** | PN | PN | PN | PY | PN | PY | PN | PN | PN | PY |
| **2.2.4** | N | Y | N | Y | Y | Y | N | PN | Y | Y |
| **2.2.5** | Y | Y | Y | Y | Y | NI | NI | Y | NI | NI |
| **Phase 2-domain 3: data collection and study appraisal** | NI | NI | NI | L | H | NI | NI | H | H | H |
| **2.3.1** | Y | NI | NI | Y | Y | Y | Y | Y | NI | NI |
| **2.3.2** | Y | Y | Y | Y | Y | Y | Y | Y | N | Y |
| **2.3.3** | Y | Y | Y | Y | Y | Y | Y | Y | Y | Y |
| **2.3.4** | Y | Y | Y | Y | N | Y | Y | N | N | N |
| **2.3.5** | NI | NI | Y | Y | Y | NI | NI | Y | NI | NI |
| **Phase 2-domain 4: synthesis and findings** | H | H | H | H | H | H | H | H | H | H |
| **2.4.1** | PN | PN | PN | PN | PN | PN | PN | PN | PN | PN |
| **2.4.2** | Y | NI | NI | Y | NI | Y | NI | NI | NI | NI |
| **2.4.3** | Y | Y | Y | Y | Y | Y | Y | Y | N | N |
| **2.4.4** | Y | N | Y | Y | N | Y | N | N | N | N |
| **2.4.5** | N | NI | NI | N | NI | Y | N | N | NI | N |
| **2.4.6** | N | N | N | N | N | Y | N | N | N | N |
| **Phase 3 risk of bias in the review** | H | H | H | H | H | H | H | H | H | H |
| **A** | N | N | N | N | N | N | N | N | N | N |
| **B** | Y | Y | Y | Y | Y | Y | Y | Y | Y | Y |
| **C** | Y | N | N | Y | Y | Y | Y | Y | Y | Y |

Y: yes; PY: probably yes; PN: probably no; N: no; NI: no information; L: low risk; H: high risk.

# Supplementary Table S6 Results of the PRISMA checklist

| **Section** | **Topic** | **Akram (2024) (21)** | **Liu ZC (2023) (22)** | **Kuang (2022) (23)** | **Zhang (2021) (24)** | **Liu Z (2015) (25)** | **White (2014) (26)** | **Tahiri (2012) (27)** | **Cheng (2012) (28)** | **White (1999) (29)** | **Ashenden (1997) (30)** | **Y+PY%** |
| --- | --- | --- | --- | --- | --- | --- | --- | --- | --- | --- | --- | --- |
| **Title** |  |  |  |  |  |  |  |  |  |  |  |  |
|  | 1. Title | Y | Y | Y | Y | Y | Y | Y | Y | Y | Y | 100 |
| **Abstract** |  |  |  |  |  |  |  |  |  |  |  |  |
|  | 2. Structured summary | PY | PY | PY | PY | PY | PY | PY | PY | PY | PY | 100 |
| **Introduction** |  |  |  |  |  |  |  |  |  |  |  |  |
|  | 3. Rationale | Y | Y | Y | Y | Y | Y | Y | Y | Y | Y | 100 |
|  | 4. Objectives | Y | Y | Y | Y | Y | Y | Y | Y | Y | Y | 100 |
| **Methods** |  |  |  |  |  |  |  |  |  |  |  |  |
|  | 5. Eligibility criteria | PY | PY | PY | Y | PY | Y | PY | PY | PY | Y | 100 |
|  | 6. Information sources | PY | PY | PY | Y | PY | Y | PY | PY | PY | PY | 100 |
|  | 7. Search strategy | Y | N | N | Y | N | Y | N | N | N | N | 30 |
|  | 8. Selection process | Y | Y | Y | Y | Y | N | N | Y | N | N | 60 |
|  | 9. Data collection process | Y | N | N | Y | Y | Y | Y | Y | N | N | 60 |
|  | 10. Data items | PY | PY | PY | PY | PY | Y | PY | PY | PY | PY | 100 |
|  | 11. Study risk of bias assessment | PY | PY | Y | Y | Y | PY | PY | Y | PY | PY | 100 |
|  | 12. Effect measures | Y | Y | Y | Y | Y | Y | Y | Y | Y | Y | 100 |
|  | 13. Synthesis methods | PY | PY | PY | PY | PY | Y | PY | PY | PY | PY | 100 |
|  | 14. Reporting bias assessment | Y | Y | Y | Y | Y | Y | Y | Y | N | N | 80 |
|  | 15. Certainty assessment | N | Y | N | Y | Y | Y | Y | N | N | N | 50 |
| **Results** |  |  |  |  |  |  |  |  |  |  |  |  |
|  | 16. Study selection | PY | PY | PY | Y | PY | Y | PY | PY | N | N | 80 |
|  | 17. Study characteristics | Y | Y | Y | Y | Y | Y | Y | Y | Y | Y | 100 |
|  | 18. Risk of bias in studies | Y | Y | Y | Y | N | Y | Y | Y | N | Y | 80 |
|  | 19. Results of individual  studies | Y | Y | Y | Y | Y | Y | PY | Y | PY | PY | 70 |
|  | 20. Results of Synthesis | PY | PY | PY | Y | N | Y | PY | PY | PY | PY | 90 |
|  | 21. Reporting biases | Y | N | Y | N | Y | Y | N | Y | N | N | 50 |
|  | 22. Certainty of evidence | N | N | N | Y | Y | Y | N | N | N | N | 30 |
| **Discussion** |  |  |  |  |  |  |  |  |  |  |  |  |
|  | 23. Discussion | PY | PY | PY | Y | PY | Y | PY | PY | PY | PY | 100 |
| **Other information** |  |  |  |  |  |  |  |  |  |  |  |  |
|  | 24. Registration and protocol | Y | N | N | Y | N | Y | N | N | N | N | 30 |
|  | 25. Support | Y | N | N | Y | N | Y | Y | N | Y | N | 50 |
|  | 26. Competing interests | N | N | N | Y | N | Y | Y | N | N | N | 30 |
|  | 27. Availability of data, code, and other materials | Y | N | N | Y | N | Y | N | N | N | N | 30 |

**Abbreviations:** Y: yes; N: no; PY: partial yes.

# Supplementary Table S7 GRADE quality grading of included SRs

| **Author (year)** | **Outcomes** | **Intervention vs. Control** | **Number of studies (total sample)** | **Pooled effect size** | **I^2^ (%)** | **Limitations** | **Inconsistency** | **Indirectness** | **Imprecision** | **Publication bias** | **Quality of evidence** |
| --- | --- | --- | --- | --- | --- | --- | --- | --- | --- | --- | --- |
| Akram (2024) (21) | FTND | LA/LA + BT vs. BT | 4(264) | OR=-0.60 (-0.97, -0.23) | 16 | Serious | Not serious | Not serious | Serious | None | L⊕⊕⊖⊖^1,3^ |
|  | FR at the end of treatment | LA vs. SA | 3(697) | OR=0.23 (0.04, 1.24) | 95 | Serious | Serious | Not serious | Serious | None | CL⊕⊖⊖⊖^1,2,3^ |
|  | FR at 3-month follow-up | LA vs. SA | 3(637) | OR=0.19 (0.02, 1.62) | 95 | Serious | Serious | Not serious | Serious | None | CL⊕⊖⊖⊖^1,2,3^ |
| Liu ZY (2023) (22) | AR (Undefined) | Acupuncture vs. Medicine | 7(658) | RR=0.95 (0.82, 1.10) | 0 | Serious | Not serious | Not serious | Serious | None | L⊕⊕⊖⊖^1,3^ |
|  |  | Acupuncture vs. SA | 6(659) | RR=1.18 (0.66, 2.11) | 63 | Serious | Serious | Not serious | Serious | None | CL⊕⊖⊖⊖^1,2,3^ |
|  |  | Acupuncture vs. BT | 2(110) | RR=2.19 (1.39, 3.45) | 0 | Serious | Not serious | Not serious | Serious | None | L⊕⊕⊖⊖^1,3^ |
|  | Relieving short-term withdrawal symptoms | Acupuncture vs. Medicine | 3(231) | RR=1.01 (0.95, 1.07) | 0 | Serious | Not serious | Not serious | Serious | None | L⊕⊕⊖⊖^1,3^ |
|  | FTND | Acupuncture vs. Medicine | 7(608) | MD=0.16 (-0.08, 0.41) | 0 | Serious | Not serious | Not serious | Serious | None | L⊕⊕⊖⊖^1,3^ |
|  |  | Acupuncture vs. SA | 4(367) | MD=-1.58 (-3.44, 0.27) | 93 | Serious | Serious | Not serious | Serious | None | CL⊕⊖⊖⊖^1,2,3^ |
|  |  | Acupuncture vs. BT | 3(182) | MD=-1.41 (-1.74, -1.08) | 86 | Serious | Serious | Not serious | Serious | None | CL⊕⊖⊖⊖^1,2,3^ |
|  | MNWS | Acupuncture vs. NRT | 4(301) | MD=0.12 (-1.11, 1.35) | 0 | Serious | Not serious | Not serious | Serious | None | L⊕⊕⊖⊖^1,3^ |
|  |  | Acupuncture vs. SA | 2(327) | MD=-4.88 (-5.21, -4.55) | 0 | Serious | Not serious | Not serious | Serious | None | L⊕⊕⊖⊖^1,3^ |
|  |  | Acupuncture vs. BT | 1(50) | MD=-4.28 (-5.31, -3.25) | - | Serious | Not serious | Not serious | Serious | None | L⊕⊕⊖⊖^1,3^ |
|  | QSU | Acupuncture vs. NRT | 2(205) | MD=-0.30 (-2.78, 2.18) | 0 | Serious | Not serious | Not serious | Serious | None | L⊕⊕⊖⊖^1,3^ |
|  | HAMD | Acupuncture vs. NRT | 1(74) | MD=0.76 (-1.54, 3.06) | - | Serious | Not serious | Not serious | Serious | None | L⊕⊕⊖⊖^1,3^ |
|  | HSI | Acupuncture vs. NRT | 5(512) | MD=0.11 (-0.13, 0.36) | 0 | Serious | Not serious | Not serious | Serious | None | L⊕⊕⊖⊖^1,3^ |
|  | Adverse event incidence | Acupuncture vs. Medicine/SA | 7(583) | RR=0.72 (0.42, 1.22) | 19 | Serious | Not serious | Not serious | Serious | None | L⊕⊕⊖⊖^1,3^ |
| Kuang (2022) (23) | AR at the end of treatment | Acupuncture vs. SA/NRT/BT | 12(1457) | RR=1.29 (0.94, 1.78) | 77 | Serious | Serious | Not serious | Serious | None | CL⊕⊖⊖⊖^1,2,3^ |
|  |  | AACP vs. SA/NRT | 5(668) | RR=1.02 (0.60, 1.73) | 77 | Serious | Serious | Not serious | Serious | None | CL⊕⊖⊖⊖^1,2,3^ |
|  | AR in Short to mid-term (1-6 months) | Acupuncture vs. SA/NRT/BT | 10(1277) | RR=1.49 (1.02, 2.17) | 76 | Serious | Serious | Not serious | Not serious | Reporting bias | CL⊕⊖⊖⊖^1,2,4^ |
|  | FTND | Acupuncture vs. SA/NRT | 8(1060) | MD=-0.34 (-1.10, -0.42) | 84 | Serious | Serious | Not serious | Serious | None | CL⊕⊖⊖⊖^1,2,3^ |
|  |  | AACP vs. NRT | 2(400) | MD=0.54 (-0.07, -1.15) | 0 | Serious | Not serious | Not serious | Serious | None | L⊕⊕⊖⊖^1,3^ |
|  | MWNS | Acupuncture vs. SA/NRT | 6(808) | MD=-1.05 (-3.61, 1.52) | 96 | Serious | Serious | Not serious | Serious | None | CL⊕⊖⊖⊖^1,2,3^ |
|  |  | AACP vs. NRT | 1(200) | MD=1.09 (-0.85, 3.03) | - | Serious | Not serious | Not serious | Serious | None | L⊕⊕⊖⊖^1,3^ |
| Zhang (2021) (24) | AR in short-term (1-3 months) | AACP vs. SA/BT | 8(637) | RR=1.41 (1.04, 1.91) | 31 | Serious | Not serious | Not serious | Not serious | None | M⊕⊕⊕⊖^1^ |
|  |  | IAN vs. SA | 2(181) | RR=3.49 (0.40, 30.59) | 88 | Serious | Serious | Not serious | Serious | None | CL⊕⊖⊖⊖^1,2,3^ |
|  | AR in mid-term (3-6 months) | AACP vs. SA/BT | 8(749) | RR=1.63 (1.27, 2.09) | 10 | Serious | Not serious | Not serious | Not serious | None | M⊕⊕⊕⊖^1^ |
|  |  | TEAS vs. SA/BT | 3(325) | RR=1.58 (1.10, 2.27) | 51 | Serious | Serious | Not serious | Serious | None | CL⊕⊖⊖⊖^1,2,3^ |
|  |  | LA vs. SA | 2(427) | RR=2.98 (0.24, 37.81) | 96 | Serious | Serious | Not serious | Serious | None | CL⊕⊖⊖⊖^1,2,3^ |
|  | AR in long-term (≥6 months) | AACP vs. SA/BT | 2(74) | RR=1.85 (0.59, 5.82) | 14 | Serious | Not serious | Not serious | Serious | None | L⊕⊕⊖⊖^1,3^ |
|  |  | TEAS vs. SA/BT | 1(76) | RR=0.50 (0.05, 5.28) | - | Serious | Not serious | Not serious | Serious | None | L⊕⊕⊖⊖^1,3^ |
|  | Relieving short-term withdrawal symptoms | AACP vs. BT/SA | 4(180) | MD=-2.68 (-5.34, -0.03) | 83 | Serious | Serious | Not serious | Serious | None | CL⊕⊖⊖⊖^1,2,3^ |
|  | FTND in short-term | AACP vs. SA | 4(373) | MD=-0.32 (-1.72, 1.08) | 93 | Serious | Serious | Not serious | Serious | None | CL⊕⊖⊖⊖^1,2,3^ |
|  | FTND in mid-term | AACP vs. SA | 2(250) | MD=-0.85 (-4.00, 2.29) | 97 | Serious | Serious | Not serious | Serious | None | CL⊕⊖⊖⊖^1,2,3^ |
|  |  | TEAS vs. SA/BT | 2(247) | MD=-0.30 (-0.90, 1.49) | 51 | Serious | Serious | Not serious | Serious | None | CL⊕⊖⊖⊖^1,2,3^ |
|  | eCO level in short-term | TEAS vs. SA | 2(124) | MD=-0.64 (-6.59, 5.31) | 66 | Serious | Serious | Not serious | Serious | None | CL⊕⊖⊖⊖^1,2,3^ |
|  |  | AACP vs. SA | 1(70) | MD=-0.13 (-0.85, 0.59) | - | Serious | Not serious | Not serious | Serious | None | L⊕⊕⊖⊖^1,3^ |
|  | The relapse rate in short-term | TEAS vs. SA | 1(51) | RR=0.82 (0.64, 1.06) | - | Serious | Not serious | Not serious | Serious | None | L⊕⊕⊖⊖^1,3^ |
|  | Adverse event incidence | AACP vs. SA/BT | 4(240) | RR=2.51 (0.24, 26.59) | 70 | Serious | Serious | Not serious | Serious | None | CL⊕⊖⊖⊖^1,2,3^ |
| Liu Z (2015) (25) | AR in short-term (Undefined) | Acupuncture vs. NRT/SA/BT | 23(3006) | RR=1.48 (1.18, 1.84) | 61 | Serious | Serious | Not serious | Not serious | Reporting bias | CL⊕⊖⊖⊖^1,2,4^ |
|  | Abstinence rate in long-term (Undefined) | Acupuncture vs. NRT/SA/BT | 12(1884) | RR=1.40 (0.90, 2.17) | 51 | Serious | Serious | Not serious | Serious | None | CL⊕⊖⊖⊖^1,2,3^ |
|  | DCC | Acupuncture vs. NRT/SA/BT | 7(733) | MD=4.35 (2.03, 6.66) | 97 | Serious | Serious | Not serious | Not Serious | None | L⊕⊕⊖⊖^1,2^ |
|  | Cotinine levels in the blood | Acupuncture vs. SA/BT | 3(182) | SMD=0.73 (-0.72, 2.19) | 95 | Serious | Serious | Not serious | Serious | None | CL⊕⊖⊖⊖^1,2,3^ |
|  | FTND | Acupuncture vs. SA | 3(227) | MD=2.46 (1.96, 2.97) | 96 | Serious | Serious | Not serious | Serious | None | CL⊕⊖⊖⊖^1,2,3^ |
|  | MNWS | Acupuncture vs. SA | 2(205) | MD=0.70 (-1.97, 3.38) | 0 | Serious | Not serious | Not serious | Serious | None | L⊕⊕⊖⊖^1,3^ |
|  | BDI | Acupuncture vs. SA | 1(47) | MD=-0.30 (-4.63, 4.03) | - | Serious | Not serious | Not serious | Serious | None | L⊕⊕⊖⊖^1,3^ |
| White (2014) (26) | AR in short-term (≤ 6 weeks after quit date) | Acupuncture vs. SA | 19(2588) | RR=1.22 (1.08, 1.38) | 46 | Serious | Not serious | Not serious | Not serious | Reporting bias | L⊕⊕⊖⊖^1,4^ |
|  |  | Acupuncture vs. NRT | 2(914) | RR=0.76 (0.59, 0.98) | 0 | Serious | Not serious | Not serious | Not serious | None | M⊕⊕⊕⊖^1^ |
|  |  | Acupuncture vs. BT | 3(396) | RR=0.95 (0.72, 1.26) | 43 | Serious | Not serious | Not serious | Serious | None | L⊕⊕⊖⊖^1,3^ |
|  |  | AACP vs. SA | 3(253) | RR=2.54 (1.27, 5.08) | 0 | Serious | Not serious | Not serious | Serious | None | L⊕⊕⊖⊖^1,3^ |
|  |  | IN vs. SA | 7(659) | RR=1.24 (0.91, 1.69) | 0 | Serious | Not serious | Not serious | Serious | None | L⊕⊕⊖⊖^1,3^ |
|  |  | IAN/AACP vs. SA | 7(496) | RR=2.73 (1.78, 4.18) | 0 | Serious | Not serious | Not serious | Serious | None | L⊕⊕⊖⊖^1,3^ |
|  |  | TEAS vs. SA | 6(634) | RR=1.13 (0.87, 1.46) | 0 | Serious | Not serious | Not serious | Serious | None | L⊕⊕⊖⊖^1,3^ |
|  | AR in long-term (≥ 6 months after quit date) | Acupuncture vs. WL/NT | 3(393) | RR=1.79 (0.98, 3.28) | 57 | Serious | Serious | Not serious | Serious | None | CL⊕⊖⊖⊖^1,2,3^ |
|  |  | Acupuncture vs. SA | 11(1892) | RR=1.10 (0.86, 1.40) | 23 | Serious | Not serious | Not serious | Serious | None | L⊕⊕⊖⊖^1,3^ |
|  |  | Acupuncture vs. NRT | 2(914) | RR=0.64 (0.42, 0.98) | 0 | Serious | Not serious | Not serious | Not serious | None | M⊕⊕⊕⊖^1^ |
|  |  | Acupuncture vs. BT | 3(396) | RR=1.34 (0.80, 2.24) | 64 | Serious | Serious | Not serious | Serious | None | CL⊕⊖⊖⊖^1,2,3^ |
|  |  | IN vs. SA | 4(446) | RR=1.20 (0.62, 2.32) | 0 | Serious | Not serious | Not serious | Serious | None | L⊕⊕⊖⊖^1,3^ |
|  |  | IAN/AACP vs. SA | 2(124) | RR=9.45 (1.26, 70.92) | 0 | Serious | Not serious | Not serious | Serious | None | L⊕⊕⊖⊖^1,3^ |
|  |  | TEAS vs. SA | 2(405) | RR=0.87 (0.61, 1.23) | 46 | Serious | Not serious | Not serious | Serious | None | L⊕⊕⊖⊖^1,3^ |
| Tahiri (2012) (27) | AR in long-term (≥ 6 months) | Acupuncture vs. SA | 6(825) | OR=3.53 (1.03, 12.07) | 85 | Serious | Serious | Not serious | Not serious | None | L⊕⊕⊖⊖^1,2^ |
| Cheng (2012) (28) | AR at the end of treatment | Acupuncture vs. SA/WL/NRT/BT | 17(4160) | RR=1.24 (1.07, 1.43) | 33 | Serious | Not serious | Not serious | Not serious | None | M⊕⊕⊕⊖^1^ |
|  | AR at 3-month follow-up | Acupuncture vs. SA/BT/PBO/WL | 9(2164) | RR=1.70 (1.17, 2.46) | 62 | Serious | Serious | Not serious | Not serious | None | L⊕⊕⊖⊖^1,2^ |
|  | AR at 6-month follow-up | Acupuncture vs. SA/BT/PBO/WL | 9(1951) | RR=1.79 (1.13, 2.82) | 58 | Serious | Serious | Not serious | Not serious | None | L⊕⊕⊖⊖^1,2^ |

**Notes:**
1. Downgraded if large biases such as poor randomization, lack of blinding, or issues with distributive hiding were present in the studies.
2. Downgraded if there was significant variability between study results, indicated by a P-value smaller than 0.1 or an I² statistic greater than 50%.
3. Downgraded if the sample size was small, fewer than 500 participants, or if the confidence intervals were wide, particularly if they included the null line where p is greater than 0.05.
4. Downgraded with caution. Publication bias was only considered when at least 10 studies were included and if relevant bias assessments, such as funnel plot asymmetry or Egger’s test, were conducted. No downgrading was applied when fewer than 10 studies were included or when publication bias testing was not performed.
**Abbreviations:** MA, manual acupuncture; EA, electroacupuncture; AACP, auricular acupressure; IAN, indwelling auricular needle; TEAS, transcutaneous electrical acupoint stimulation; LA, laser acupuncture; ACE, acupoint catgut embedding; FN, fire needling; BT, behavioral therapy; SA, sham acupuncture; WL, waiting list; PBO, placebo; AR, abstinence rate; DCC, Daily cigarette consumption; FTND, Fagerström Test for Nicotine Dependence; MNWS, Minnesota Nicotine Withdrawal Scale; QSU, Brief Questionnaire of Smoking Urges; HAMD, Hamilton Depression Rating Scale; HSI, Heaviness of Smoking Index; eCO, exhaled CO level; BDI, Beck Depression Inventory; FA, smoking cessation failure rate. NRT, nicotine replacement therapy; CL, critically low; L, low; M, moderate.

# Supplementary Table S8 Results of the Egger’s test

| **Outcome** | **Bias Estimate (SE)** | **t (df)** | **p-value** | **τ²** | **Interpretation** |
| --- | --- | --- | --- | --- | --- |
| Acupuncture vs. SA for short-term abstinence | 0.4993 (0.6044) | 0.83 (28) | 0.416 | 2.998 | No significant publication bias |
| Acupuncture vs. SA for long-term abstinence | 0.8296 (0.9636) | 0.86 (12) | 0.406 | 3.8831 | No significant publication bias |
| Acupuncture vs. NRT for short-term abstinence | -0.3752 (0.4453) | -0.84 (6) | 0.432 | 0.1516 | No significant publication bias |
| Acupuncture vs. NRT for short-term abstinence | -0.8829 (0.9082) | -0.97 (4) | 0.386 | 0.4769 | No significant publication bias |
| Acupuncture vs. BT for short-term abstinence | 1.6770 (2.3787) | 0.71 (2) | 0.554 | 1.8195 | No significant publication bias (low power) |
| Acupuncture vs. BT for long-term abstinence | -1.9840 (3.7243) | -0.53 (2) | 0.648 | 3.2759 | No significant publication bias (low power) |
| Acupuncture vs. WL for short-term abstinence | 13.5347 (9.5571) | 1.42 (2) | 0.292 | 3.0634 | No significant publication bias (low power) |
| Acupuncture vs. WL for long-term abstinence | 1.9643 (1.1196) | 1.75 (2) | 0.221 | 0.9091 | No significant publication bias (low power) |

**Abbreviations:** SA, sham acupuncture; WL, waiting list; BT, behavioral therapy; NRT, nicotine replacement therapy.
